# A contemporary baseline of Madagascar’s coral assemblages: Reefs with high coral diversity, abundance, and function associated with marine protected areas

**DOI:** 10.1371/journal.pone.0275017

**Published:** 2022-10-20

**Authors:** Mahery Randrianarivo, François Guilhaumon, Johanès Tsilavonarivo, Andriamanjato Razakandrainy, Jacques Philippe, Radonirina Lebely Botosoamananto, Lucie Penin, Gildas Todinanahary, Mehdi Adjeroud

**Affiliations:** 1 Institut Halieutique et des Sciences Marines, Université de Toliara, Toliara, Madagascar; 2 ENTROPIE, Université de La Réunion, IRD, CNRS, IFREMER, Université de la Nouvelle-Calédonie, La Réunion, France; 3 Centre National de Recherches Océanographiques, Nosy-Be, Madagascar; 4 Laboratoire d’Excellence “CORAIL”, Paris, France; 5 ENTROPIE, Université de La Réunion, IRD, CNRS, IFREMER, Université de la Nouvelle-Calédonie, Perpignan, France; 6 UAR 3278 CRIOBE EPHE-UPVD-CNRS, PSL Université Paris, Perpignan, France; Secretariat of the Pacific Community, NEW CALEDONIA

## Abstract

Madagascar is a major hotspot of biodiversity in the Western Indian Ocean, but, as in many other regions, coral reefs surrounding the island confront large-scale disturbances and human-induced local stressors. Conservation actions have been implemented with encouraging results for fisheries, though their benefit on coral assemblages has never been rigorously addressed. In this context, we analyzed the multiscale spatial variation of the composition, generic richness, abundance, life history strategies, and cover of coral assemblages among 18 stations placed at three regions around the island. The potential influences of marine protected areas (MPAs), algal cover, substrate rugosity, herbivorous fish biomass, and geographic location were also analyzed. Our results highlight the marked spatial variability, with variation at either or both regional and local scales for all coral descriptors. The northeast coastal region of Masoala was characterized by the high abundance of coral colonies, most notably of the competitive *Acropora* and *Pocillopora* genera and stress-tolerant taxa at several stations. The southwest station of Salary Nord was distinguished by lower abundances, with depauperate populations of competitive taxa. On the northwest coast, Nosy-Be was characterized by higher diversity and abundance as well as by high coral cover (~42–70%) recorded at unfished stations. Results clearly underline the positive effects of MPAs on all but one of the coral descriptors, particularly at Nosy-Be where the highest contrast between fished and unfished stations was observed. Biomass of herbivorous fishes, crustose coralline algae cover, and substrate rugosity were also positively related to several coral descriptors. The occurrence of reefs with high diversity, abundance, and cover of corals, including the competitive *Acropora*, is a major finding of this study. Our results strongly support the implementation of locally managed marine areas with strong involvement by primary users, particularly to assist in management in countries with reduced logistic and human resources such as Madagascar.

## Introduction

More than 850 million people from over 100 countries rely on the exceptional biodiversity of coral reefs, providing crucial economic, cultural, social, and aesthetic goods and services [1, 2]. These reefs are mostly supported by small colonial, calcifying organisms, the hermatypic scleractinian corals, which create complex three-dimensional habitats offering a variety of shelter and food for thousands of organisms [3, 4]. Coral assemblages often exhibit a marked spatial heterogeneity from local to geographic scales (i.e., from within and among reef habitats to among regions and ocean basins), reflecting the contrasting life history strategies and functional traits of coral species as well as the biological and physical processes that influence their biology and vary in frequency, intensity, and spatial scale [5–9]. Since corals are particularly sensitive to changes in environmental conditions, reef ecosystems are highly vulnerable to both chronic and acute stressors and may change rapidly in their structure and functioning [10–13].

There is concern that the frequency and severity of large-scale disturbances, such as coral bleaching events associated to thermal stress, cyclones, or outbreaks of the coral predator *Acanthaster* spp. in the Indo-Pacific, have increased over the last four decades [4, 14, 15]. Humans have further contributed to this declining trend by contributing multiple, direct anthropogenic disturbances that kill reef organisms, such as overharvesting of reef organisms, destructive fishing methods, uncontrolled tourism and recreational impacts, and increased sedimentation and pollution associated with dredging, coastal development, deforestation, and intensive agriculture [4, 16, 17]. In response to these threats, coral reefs have been severely impacted by widespread mortalities of foundation species and degraded habitat [18–20], in a number of cases undergoing a striking phase shift involving the replacement of corals by macroalgae or other non-reef-building benthic organisms, an undesirable state providing fewer goods and services to human populations [21].

In the context of this “coral reef crisis”, evaluating the vulnerability, adaptability, and resilience of reef communities and their dependent human societies is urgently needed [4, 22, 23]. Encouragingly, management and conservation actions have already been taken to preserve the diversity, structure, and functions of coral reefs and to maintain the goods and services they provide to humans [24–26]. The Marine Protected Area (MPA) has been one of the most common tools to support the health of coral reefs and to enhance their resistance and resilience to disturbances [27–31]. Although the capacity of MPAs to maintain fisheries productivity and sustainable livelihoods has been demonstrated in many cases [32–36] conflicting results and uncertainties regarding their effectiveness in protecting the transformed reefs that are expected in future decades have also been highlighted [6, 26, 32, 37–40]. For corals, management of fishing activities within MPAs is intended to maintain a sufficient herbivory rate to remove algae that are competitive with corals, in turn enhancing coral recruitment [31, 41–43]. However, this mechanism is not always observed, and a lack of positive effects of MPAs on coral replenishment capacities has been recorded [44, 45]. Among the major drivers of MPA effectiveness are the duration of protection, size, and connectivity, together with the type and compliance of regulation measures [29, 32, 46–49].

Madagascar is one of the largest islands in the world, with a coastline extending over ~14° of latitude (11° 47’ to 25° 35’ S) and, with ~2400 km^2^ of coral reefs, is a major biodiversity hotspot in the Western Indian Ocean [50–52]. The high diversity of reef organisms, including ~380 scleractinian coral species, is partly linked to the size, morphological diversity, and contrasting environmental conditions of these reefs [6, 53–57]. Studies on the diversity and structure of coral assemblages have taken place since the 1960s with the establishment of the marine research center at Toliara on the southwest coast [58]. However, most of these studies have been restricted to either local [59–65] or regional [38, 55] scales, or have focused on documenting the general health status of these reefs [53, 55, 66–68]. In contrast, no multiscale analysis of spatial patterns in coral assemblages has been conducted, and the drivers of such variability remain poorly understood.

As most of the world’s coral reefs, those of Madagascar have confronted large-scale disturbances and human-induced local stressors, most notably bleaching events, sedimentation, and overfishing [56, 67, 69–71]. Consequently, coral assemblages have declined since the 1980s at several reefs around the island, as documented along the Great Reef of Toliara [61–63], though a return to a healthier coral community has recently been documented for this reef [65]. In response to the overall degradation of coral reefs, the Malagasy authorities have implemented mitigation actions, resolutely investing in the establishment of MPAs since 1989, with the creation of the first one, the Mananara Nord Biosphere Reserve on the east coast. Twenty-two MPAs currently cover an area of 14,451 km², representing 1.26% of the Exclusive Economic Zone [72, 73]. Involvement of primary users, most notably fishermen and sea farmers, have been encouraged through the implantation of Locally Managed Marine Areas (LMMA) that have shown some success in effectively managing fisheries [35, 74, 75]. However, the effects of these MPAs on coral assemblages have not been rigorously examined, leaving the benefit and effects of such a management tool on the overall reef biodiversity, functions, and services difficult to assess in Madagascar [35, 75].

In this context, the aim of this study was to analyze the spatial variation of coral assemblages at multiple scales around Madagascar. Composition, generic richness, abundance, life history strategies, and cover of coral assemblages were compared among 18 stations located at three regions around the island. We analyzed the potential influence of several environmental factors, including algal cover, substrate rugosity, herbivorous fish biomass, and geographic location on the spatial patterns of coral variables. By comparing stations in unfished areas (MPAs) and stations where fishing is allowed, we also investigated the effect of fishing protection level on coral community structure. Although this study is a snapshot in a highly dynamic system, the data set and questions addressed in this study provide a valuable contemporary baseline to understand the drivers of coral community structure and to monitor future changes of these reefs, and may also help identify effective conservation and management actions.

## Materials and methods

### Study area and sampling strategy

**Fig 1:**
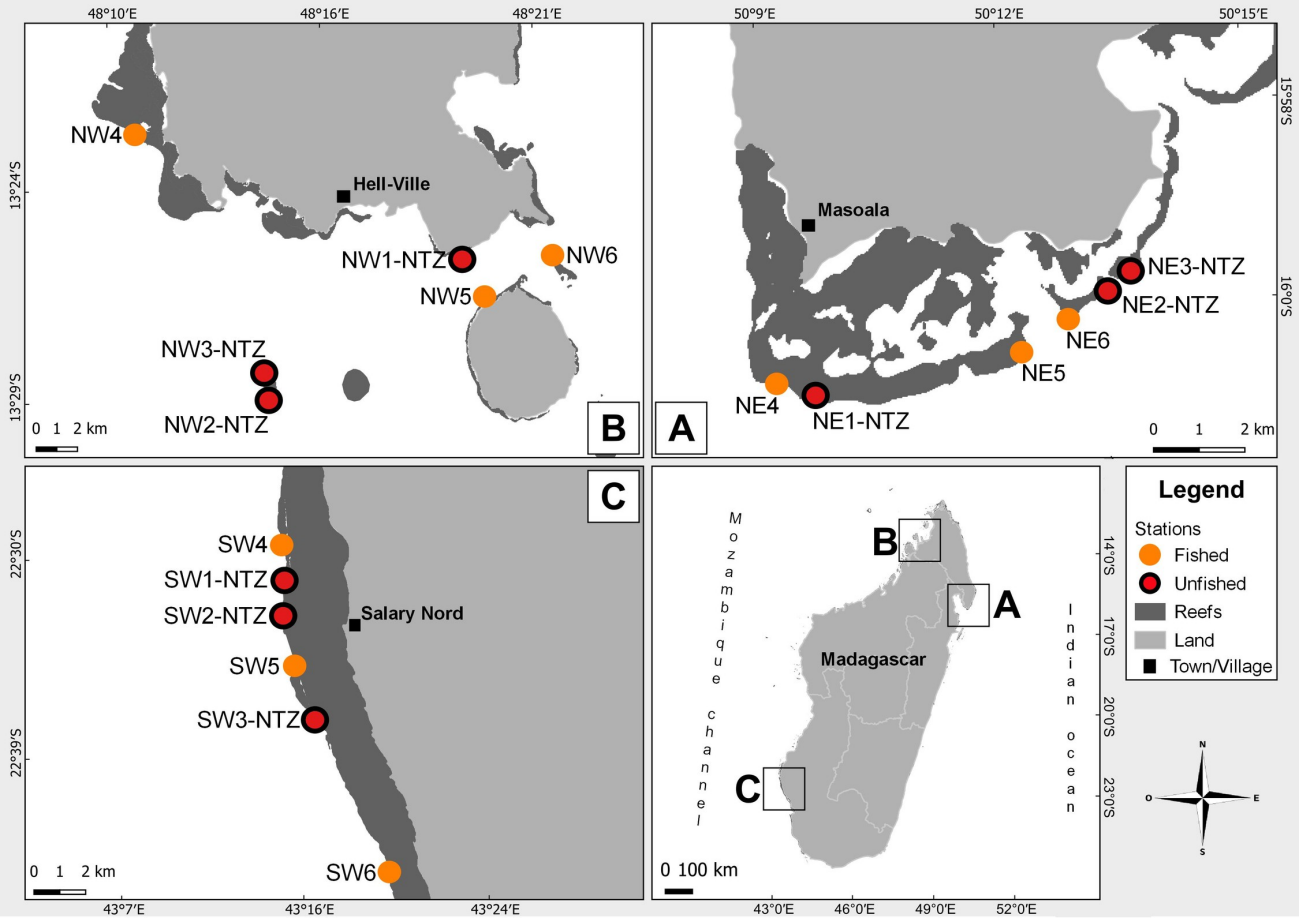
Location of the 18 sampling stations in the three regions around Madagascar. Masoala (A) is located in the northeast coast, Nosy-Be (B) in the northwest, and Salary Nord (C) in the southwest. See Materials and Methods section for station codes.

Fieldwork was conducted from March to October 2020 at three regions around Madagascar (Fig 1). Masoala, on the northeast coast (15°59’08” S, 50°09’27” E), is composed of fringing reefs of 0.5 to 3 km wide with several passes to the open ocean. This region includes the Masoala Marine Park, created in March 1997 (S1 Table). Nosy-Be, in the northwest (13°24’21” S, 48°16’31” E), is composed by several small islands with narrow (< 1 km) fringing reefs. This region comprises the Lokobe Marine Park and the Nosy Tanihely Marine Park, both created in September 2011. Salary Nord, along the southwest coast (22°33’17” S, 43°17’10” E), is a region with fringing reefs of 1.7 to 4.9 km wide, with a channel (~5 m depth) that allows the circulation of boats. These reefs are integrated within the Soariaka MPA, created in April 2015. In recent decades, these reefs have been impacted by the bleaching event of 1998 [70, 76], and 2016 [77] (S1 Table). The major cyclones that had a potential impact on coral assemblages are Haruna at Salary Nord in 2013, Enawo 2017 at Masoala and Nosy-Be in 2017, and Belan at Nosy-Be in 2019 [78]. However, the impact of these disturbances on coral assemblages in our three regions has not been quantified. At each region, six sampling stations were surveyed, including three stations in unfished areas and three stations in areas where fishing is allowed (Fig 1 and S1 Table). The location of our stations took into account logistical considerations (accessibility of sites), meteorological constraints during sampling, and the directives of local authorities (Madagascar National Park, Wildlife Conservation Society, and local managers). All 18 stations were located on the outer reef slope at ~10 m depth, where direct anthropogenic disturbances are lower, and where diversity and abundance of coral assemblages is generally higher compared to other reef habitats [65]. Stations codes are abbreviated as follow: the first two letters indicate the region (NE for Masoala in the northeast, NW for Nosy-Be in the northwest, and SW for Salary Nord in the southwest), the number (1 to 6) differentiates the six stations from each region, and NTZ (“No Take Zone”) is for stations in unfished areas. Field work was carried out with a research permit granted by the Malagasy Ministry of Environment and Sustainable Development (75/20/MEDD/SG/DGEF/DGNRE).

At each station, generic richness (GR) and abundance of coral assemblages (scleractinian corals and the calcareous hydrocoral *Millepora*) were estimated using three randomly replicated belt-transects of 10 m^2^ (10 × 1 m), laid parallel to depth contours and separated by ~2 m [79]. To complement the characterization of the diversity of coral assemblage composition, the Shannon diversity index (H’) was calculated at each station using log_2_ and colony abundance data for each coral genus. In order to estimate the functional trait diversity of coral assemblages, we assigned one of the four life history strategies, as defined by Darling et al. [80], to each coral colony encountered in the belt-transects: competitive, generalist, stress-tolerant, and weedy. For those coral genera that encompass species spanning several of these life history strategies, we have assigned proportional values based on life history strategy of the species typically found in the region ([81]; for example, *Pocillopora* was estimated to include 75% generalist and 25% weedy species). The percent cover of corals, macroalgae, turf algae, and crustose coralline algae (CCA) was estimated at each station using three line intercept transects (LIT) of 10 m, placed in the middle of the belt-transects. Biomass of herbivorous fishes was estimated at each station using underwater visual censuses on three randomly replicated belt-transects of 250 m² (50 × 5 m) laid parallel to depth contours and separated by ~2 m. All herbivorous fishes were identified at the species level, counted, and measured according to their total length. Abundance values were converted to kilograms of biomass per unit area of reef (kg.ha^-1^) using species-specific length-weight equations: W = a⋅L^b^, where *W* is the weight (in g), *L* is the total length (in cm), and parameters *a* and *b* are species-specific constants that have been extracted from FishBase [82]. At each station, we estimated the substrate rugosity with a visual assessment of reef topography, graded from 0 to 5 (0 = no vertical relief; 1 = low and sparse relief; 2 = low but widespread relief; 3 = moderately complex; 4 = very complex with numerous fissures and caves; 5 = exceptionally complex with numerous caves and overhangs), following Polunin & Roberts [83].

Cover of algae (macroalgae, turf, and CCA), rugosity, herbivorous fish biomass, fishing protection level (fished *vs*. unfished areas; i.e., we did not measure fishing pressure at each station), and geographic location (belonging to one of the three regions) have been recorded to estimate their potential influence on the spatial variation of coral assemblages, and will be referred to as “explanatory variables” hereafter.

### Data analysis

The spatial variation in the composition of coral assemblages was analyzed using nonmetric multidimensional scaling (nMDS), based on the Bray-Curtis dissimilarity index of the abundance of coral genera recorded at each station. The nMDS was performed using the “metaMDS” function in the “vegan” R package [84]. The potential influence of explanatory variables was analyzed with PERMANOVA on distance matrices using the “Adonis” function in the “vegan” R package [85]. Additionally, pairwise post-hoc multilevel comparisons were conducted to test for significant differences in the composition of coral assemblages between the three regions, using the “pairwise.Adonis2” function in the “pairwiseAdonis” R package [86].

We used linear mixed effect models to determine which explanatory variables were influencing the spatial variation of the coral assemblage descriptors (GR, H’, total abundance, abundance of the four life history strategies, and overall coral cover). Prior to analysis, Spearman rank correlations were calculated between all pairs of explanatory variables (cover of macroalgae, turf algae, CCA, herbivorous fish biomass, and rugosity) to identify potential collinearity between these variables. As no strong correlations (-0.7 < overall ρ < 0.7) were recorded, all our explanatory variables were considered as independent and included in the models (S1 Fig). All explanatory variables were centered to a mean of zero and scaled to a standard deviation of 1 in order to allow for direct comparisons of their effects. Linear mixed effect models were selected as they take into account the hierarchical structure of the data set: here, the non-independence of data points (transects) belonging to particular stations, in turn belonging to the same region [87]. We used a multi-model information-theoretic approach to compare the explanatory variable contributions and their most important interactions in describing the variation in coral descriptors. To ensure that all model assumptions were met and to take into account overdispersion, we modeled GR, H’, abundance and cover data using a Gaussian distribution, while abundance of the four life history strategies were modeled using a Poisson distribution. Models were fitted by maximum likelihood estimation using the “lmer” and “glmer” functions in the “lme4” R package [88]. We estimated how much of the variation in coral descriptors was explained by the variables included in the models using the marginal R² (“r.squaredGLMM” function in the “MuMIn” R package; [89]). For each coral variable, we selected the most parsimonious combinations of fixed effects by comparing models with all possible combinations of the predictor variables using the corrected Akaike’s information criterion (AICc; [90]). The subset of best models was selected as the ones with ΔAICc value < 2 (the difference between each model’s AICc and the lowest AICc) using the “dredge” function in the “MuMIn” R package. All models with a ΔAICc < 2 were considered as having similar levels of support from the data, thus belonging to the group of best models (i.e., equally parsimonious; [90]). We used Akaike weights (wAICc), derived from the AICc, to evaluate the relative likelihood of each model given the data set and the set of models considered as well as to estimate the relative importance of each explanatory variable by summing these wAICc across the models in which they were included. Akaike weights were directly interpreted as each model’s probability of being the best at explaining the data [90]. Subsequently, the effect sizes (lm coefficients) of the predictions of the individual models selected at the previous step were averaged using the Akaike weights as weighting coefficients. Finally, we investigated the differences among factor modalities in the mixed models using Tukey post-hoc tests (“emmeans” function in the “emmeans” R package; [91]). All statistical analyses were performed using R 4.1.0 [92].

## Results

### Composition of coral assemblages

**Fig 2:**
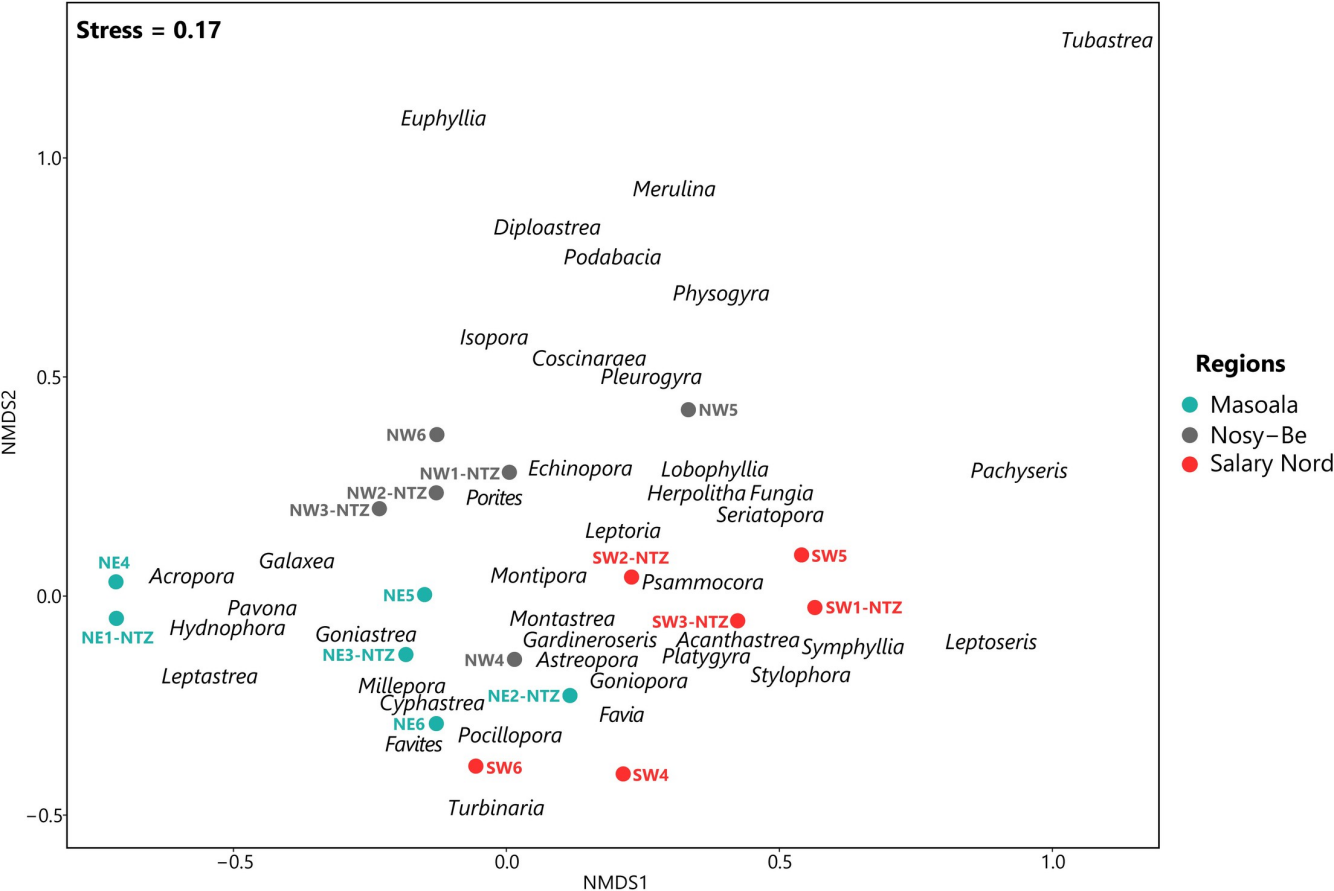
Non-metric multidimensional scaling (nMDS) showing the spatial variation in the composition and abundance of coral assemblages. Analyses are based on the Bray-Curtis dissimilarity index among the 18 stations located in the three regions. Position of stations and coral genera is given along the first two axes.

**Fig 3:**
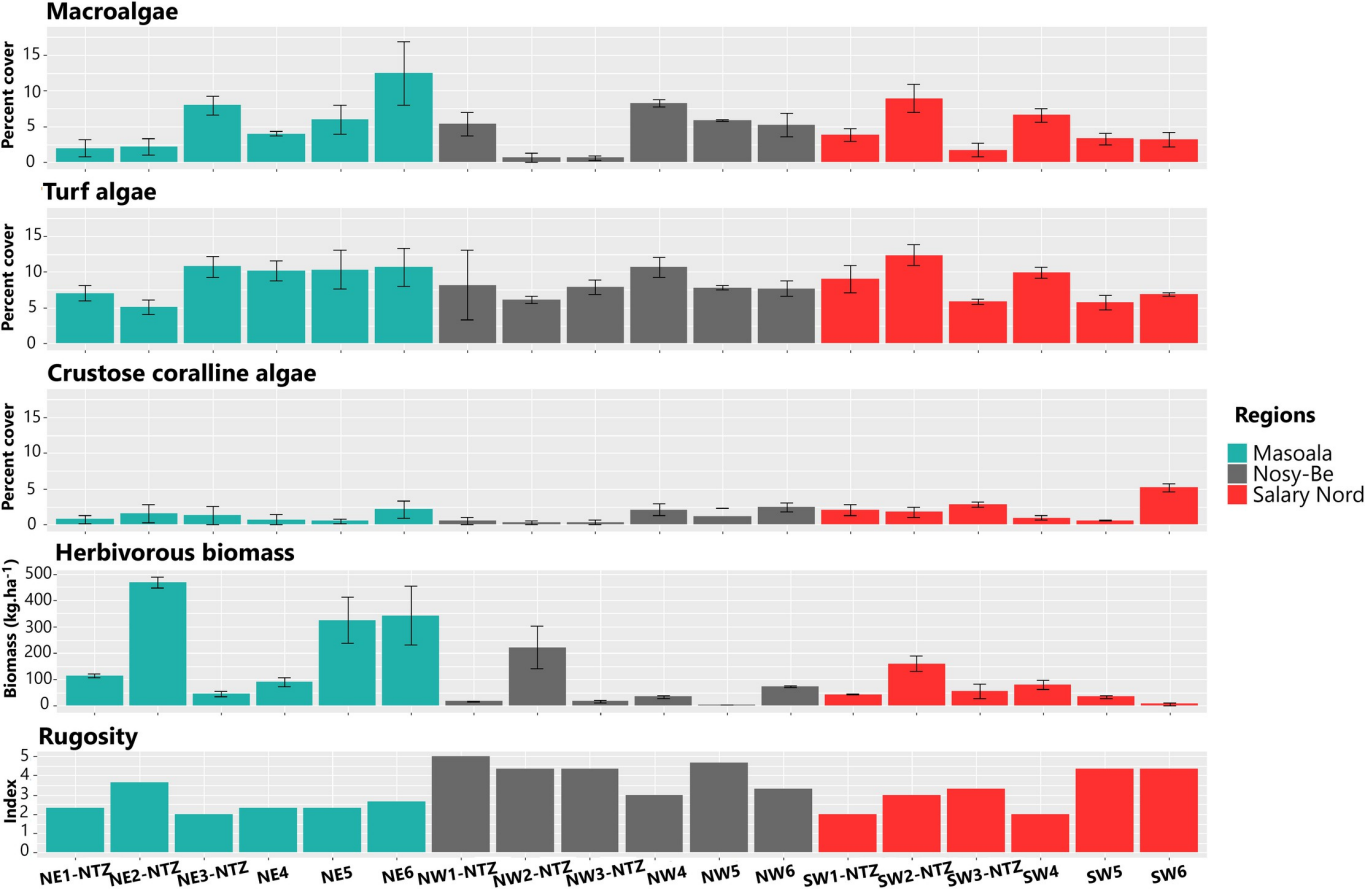
Spatial variation of explanatory variables that may influence the spatial variation of coral assemblages. Data are given for the 18 sampling stations located at the three regions. Errors bars represent standard error.

A total of 7,638 coral colonies, representing 16 families and 40 genera (30 at Masoala, 38 at Nosy-Be, and 29 at Salary Nord), were recorded at the 18 stations. The nMDS ordination showed a substantial geographical clustering in the composition of coral assemblages (Fig 2). Among the seven potential explanatory variables examined (Fig 3), geographic location (belonging to one of the three regions) was the strongest contributor to the overall dissimilarity in the composition of coral assemblages, explaining ~27% of the variance (PERMANOVA, F = 3.825, p = 0.001; R² = 0.27) and followed by rugosity, though to a lesser degree (PERMANOVA, F = 2.444, p = 0.021; R² = 0.08; Table 1). In fact, the differences in the composition of coral assemblages between regions were all significant (pairwise post-hoc tests, Masoala × Nosy-Be, F = 2.384, p = 0.013; Masoala × Salary Nord, F = 2.817, p = 0.029; Nosy-Be × Salary Nord, F = 3.380, p = 0.006). The first two axes of the nMDS differentiated the stations of Masoala, characterized by higher abundances of *Acropora*, *Pavona*, *Hydnophora*, and *Leptastrea*, from the stations of the two other regions. An exception is station NE2-NTZ, where lower abundance of *Acropora* was recorded and which was more similar to Salary Nord stations (S2 Table). Most Nosy-Be stations were located in the upper part of the nMDS plot, and were characterized by the presence of *Diploastrea*, *Euphyllia*, *Isopora*, and *Tubastrea* that were recorded only in this region (S2 Table). Station NW4 at Nosy-Be was however more similar in its composition to several Masoala and Salary Nord stations. Stations from Salary Nord, in the right portion of the nMDS plot, were characterized by higher abundance of *Leptoseris*, *Pachyseris*, and *Stylophora* at several stations (S2 Table).

**Table 1:** Summary of the PERMANOVA to examine the influence of explanatory variables in the variation of composition of coral assemblages. Analyses are based on the Bray-Curtis dissimilarity index of the abundance of coral genera recorded at the 18 stations of the three regions. Significant *P*-values (<0.05) are highlighted in bold.

Source of variation	F	*P*-value	R²
Region	3.825	**0.001**	0.276
Fishing protection level	1.498	0.176	0.054
Macroalgal cover	1.156	0.336	0.041
Turf cover	1.410	0.214	0.050
CCA cover	1.700	0.097	0.061
Herbivorous fish biomass	1.820	0.085	0.065
Rugosity	2.444	**0.023**	0.088
Region × Fishing protection level	1.493	0.131	0.107

### Generic richness

**Fig 4:**
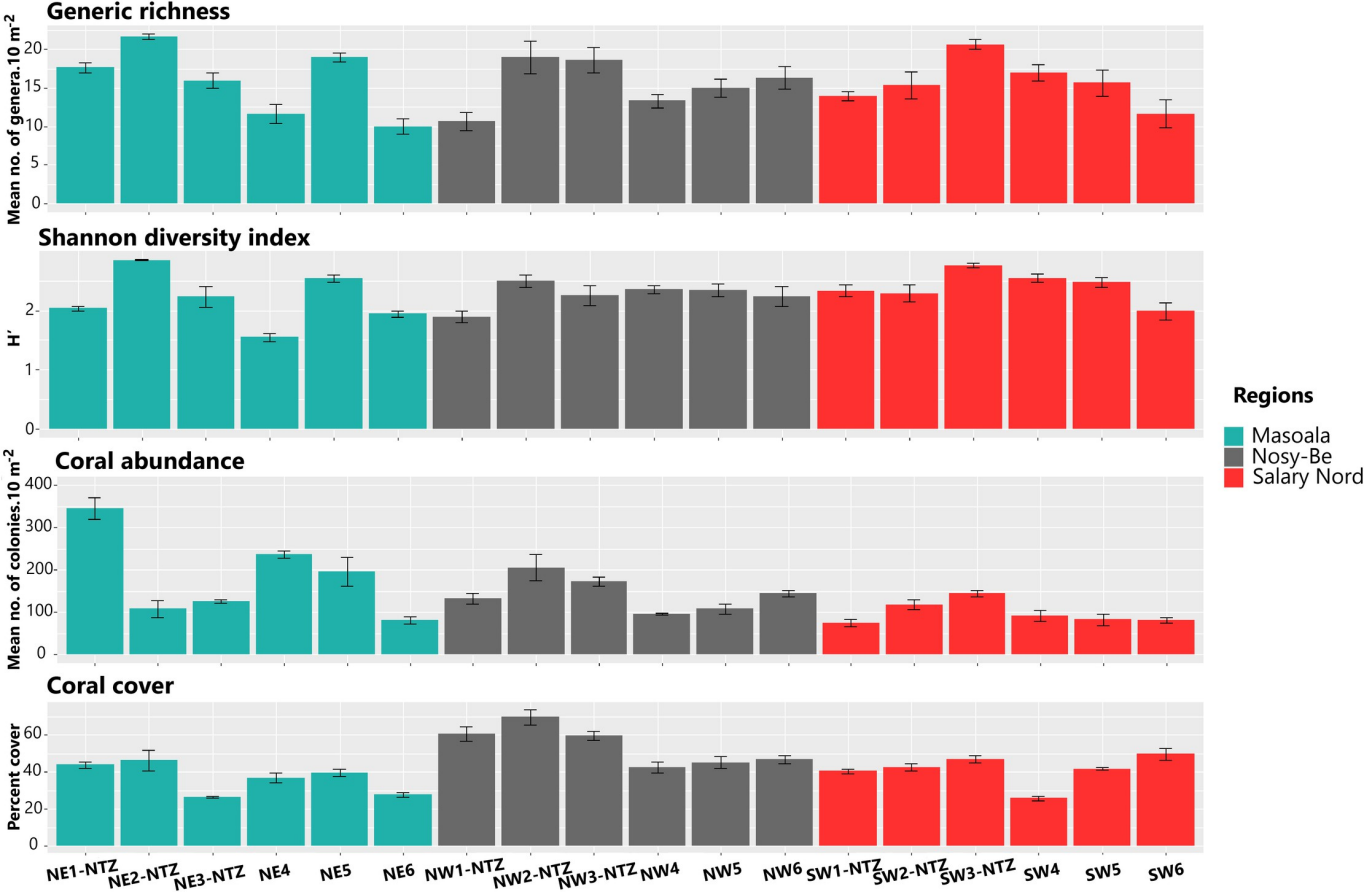
Spatial variation in generic richness, Shannon diversity index, abundance, and percent cover of coral assemblages. Data are given for the 18 sampling stations located at the three regions. Errors bars represent standard error.

Generic richness was highly variable among stations within the regions, but the range of variation was similar among the three regions, with values from 10.00 ± 1.00 genera.10 m^-2^ (mean ± se) to 21.66 ± 0.33 at Masoala, from 10.66 ± 1.20 to 19.00 ± 2.08 at Nosy-Be, and from 11.66 ± 1.76 to 20.66 ± 0.66 at Salary Nord (Fig 4 and S3 Table). The selection procedure identified four models to explain the variation in GR (ΔAIC < 2; Tables 2 and S4 and S5 and S2 and S6 Figs), with fishing protection level and herbivorous fish biomass having the strongest contribution. The biomass of herbivorous fish was included in all four models and had the maximum value of relative importance (1.000). Models indicated that the number of coral genera per 10 m^2^ increases by 1.33-fold with an increase of 1 kg.ha^-1^ in herbivorous fish biomass. Fishing protection level also had a strong influence on the variation of GR (relative importance: 0.736), with 2.55 times more coral genera per 10 m^2^ in unfished stations compared to fished ones. Cover of CCA and turf algae weakly predicted the variation in GR (relative importance: 0.191 and 0.154, respectively), with a positive coefficient for CCA (0.39) and a negative one for turf algae (-0.28). All other explanatory variables were not selected by the models to account for the variation in GR (Table 2).

**Table 2:** Summary of best linear mixed models using a Gaussian distribution to describe variation of generic richness, Shannon diversity index, coral abundance, and coral cover. FP: fishing protection level, MA: macroalgae, CCA: crustose coralline algae, HFB: herbivorous fish biomass.

Variables	Intercept	Region	FP	Rugosity	MA	Turf	CCA	HFB	Region × FP	df	Loglik	AICc	ΔAICc	wAICc	R^2^ marginal
Generic richness	14.37		×					1.353		5	-125.2	261.7	0	0.391	0.256
	15.64							1.404		4	-126.81	262.5	0.78	0.264	0.138
	14.27		×				0.393	1.215		6	-124.63	263.1	1.43	0.191	0.244
	14.4		×			-0.286		1.316		6	-124.84	263.6	1.86	0.154	0.263
*Relative importance*		0	0.736	0	0	0.154	0.191	1	0						
*Coefficient*			Unfished: 2.55			-0.28	0.39	1.33							
Shannon Index	2.273						0.075	0.079		5	1.249	8.8	0	0.313	0.104
	2.271						0.091			4	-0.112	9.1	0.27	0.274	0.062
	2.198		×				0.077	0.077		6	1.796	10.3	1.47	0.150	0.143
	2.188		×				0.092			5	0.478	10.3	1.54	0.145	0.102
	2.273			0.039			0.070	0.083		6	1.557	10.8	1.95	0.118	0.109
*Relative importance*		0	0.424	0.118	0	0	1	0.581	0						
*Coefficient*			Unfished: 0.15	0.03			0.08	0.07							
Coral abundance	141.9							26.550		4	-267.501	543.9	0	0.545	0.119
	126.5		×					26.102		5	-267.053	545.4	1.56	0.250	0.163
	142.1						-3.356	28.360		5	-267.249	545.8	1.95	0.205	0.135
*Relative importance*		0	0.250	0	0	0	0.205	1	0						
*Coefficient*			Unfished: 30.68				-3.35	26.81							
Coral cover	36.71	×	×	6.419			1.233			8	-155.308	330.0	0	0.240	0.747
	37.00	×	×	6.856						7	-157.062	330.7	0.70	0.169	0.748
	39.57		×	7.961						5	-159.756	330.8	0.85	0.157	0.648
	39.35		×	7.551			1.031			6	-158.584	331.0	1.07	0.140	0.625
	39.65		×	7.872		-0.805				6	-158.919	331.7	1.74	0.100	0.661
	37.15	×	×	6.754		-0.817				8	-156.182	331.7	1.75	0.100	0.759
	39.61	×	×	6.117			1.388		×	10	-153.232	331.8	1.87	0.094	0.789
*Relative importance*		0.603	1	1	0	0.200	0.474	0	0.094						
*Coefficient*		Nosy-Be: 6.89 Salary N: -0.67	Unfished: 8.04	7.04		-0.81	1.20		Unfished × Nosy-Be: 12.16 Unfished × Salary N:6.48						

### Shannon diversity index

The Shannon diversity index was also variable among stations within regions, particularly at Masoala, with H’ values from 1.54 ± 0.03 (mean ± se) to 2.85 ± 0.00, compared to Nosy-Be (1.89 ± 0.06 to 2.49 ± 0.06) and Salary Nord (1.98 ± 0.08 to 2.76 ± 0.02; Fig 4 and S6 Table). Five models were selected as the most parsimonious to explain variation of H’ (Tables 2 and S7 and S8 and S3 and S7 Figs). CCA cover was the most important predictor (relative importance: 1.000), followed by herbivorous fish biomass (0.581) and fishing protection level (0.424), with all three variables having a positive contribution (coefficient of 0.08, 0.07, and 0.15, respectively). Rugosity was also selected in some models, but its contribution to the spatial variation in H’ was lower (relative importance: 0.118; coefficient: 0.03).

### Coral abundance

Abundance of coral colonies was highly variable among stations within region, notably for Masoala, with 81.70 ± 8.09 to 345.66 ± 25.01 colonies.10 m^-2^ (mean ± se), compared to Nosy-Be (96.70 ± 2.03 to 205.33 ± 31.40 colonies.10 m^-2^) and Salary Nord (75.00 ± 7.77 to 144.33 ± 7.26 colonies.10 m^-2^; Fig 4 and S9 Table). Three models were selected to explain the variation in coral abundance (Tables 2 and S10 and S11 and S4 and S8 Figs). Herbivorous fish biomass had the highest relative importance (1.000), followed to a lesser degree by fishing protection level (0.250) and CCA cover (0.205). The effect of herbivorous fish biomass on coral abundance was strong and positive, with a coral abundance increase by 26.81-fold with an increase of 1 kg.ha^-1^ in herbivorous fish biomass. The models also suggested that 30.68 times more coral colonies were recorded in unfished stations, whereas the effect of CCA cover on variation in coral abundance was negative (coefficient: -3.35).

**Fig 5:**
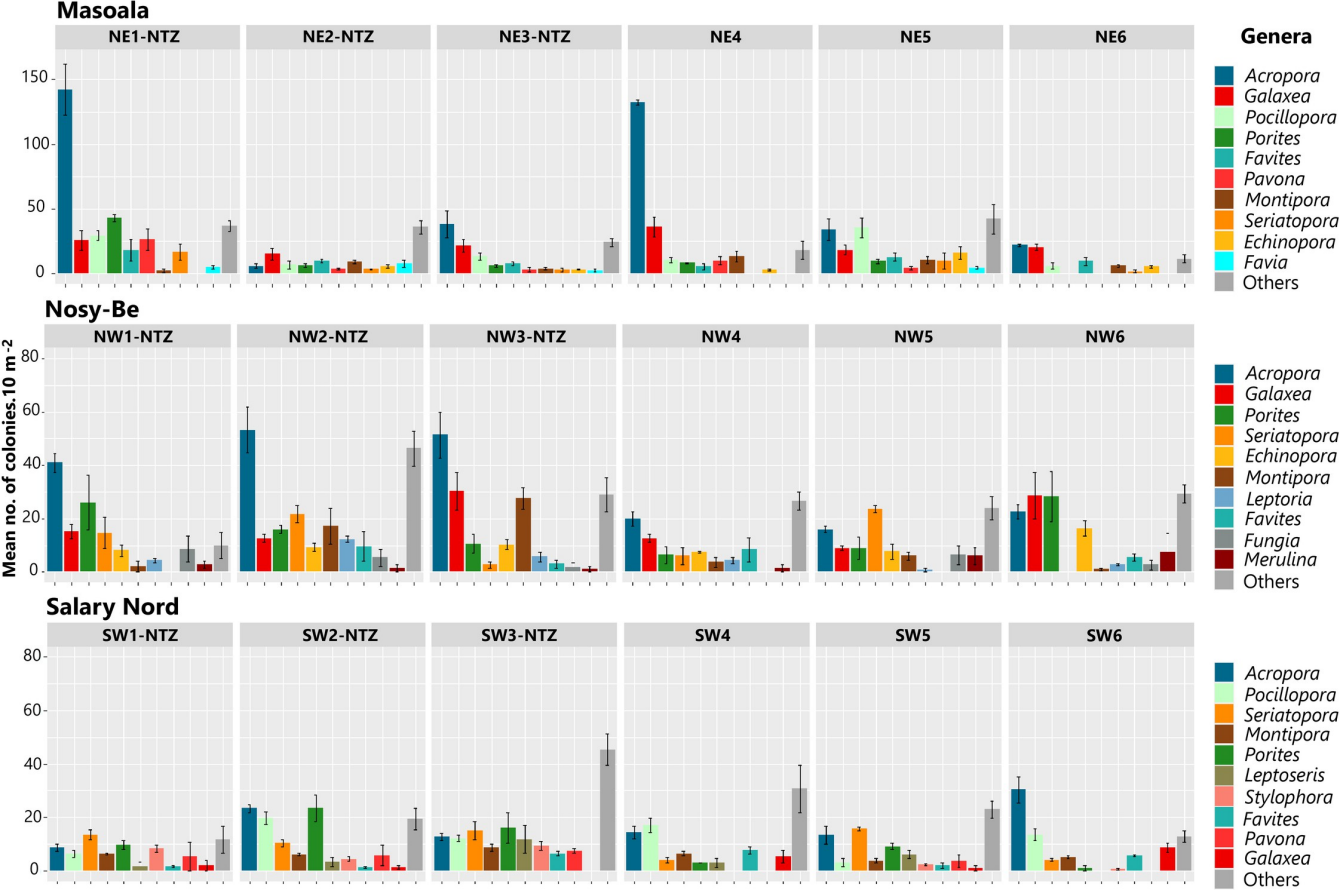
Spatial variation in the abundance of the 10 major coral genera and other less represented genera (“others”). Data are given for 18 sampling stations located at the three regions. Errors bars represent standard error.

The abundance of the 10 major coral genera at each region, as well as other, less represented genera (“others”), was highly variable among stations and regions (Fig 5). *Acropora* was the most abundant coral at eight of the 18 stations, with particularly high values at NE1-NTZ and NE4 located at Masoala and, to a lesser degree, at the unfished stations of Nosy-Be (NW1-NTZ, NW2-NTZ, NW3-NTZ). Several genera were frequently recorded at certain regions while greatly less abundant in others. This is the case for *Galaxea*, which was abundant at Masoala and Nosy-Be stations but less represented at Salary Nord, and *Pocillopora*, which was abundant at Masoala and Salary Nord but not at Nosy-Be. *Porites* and *Seriatopora* were recorded at almost all stations, being among the dominant corals at several stations (NW6 and SW2-NTZ for *Porites*, NW5, SW1-NTZ, and SW5 for *Seriatopora*). Several genera were part of the 10 dominant corals at only one of the three regions, as *Favia* at Masoala, *Leptoria*, *Fungia* and *Merulina* at Nosy-Be, and *Leptoseris* and *Stylophora* at Salary Nord (Fig 5).

**Fig 6:**
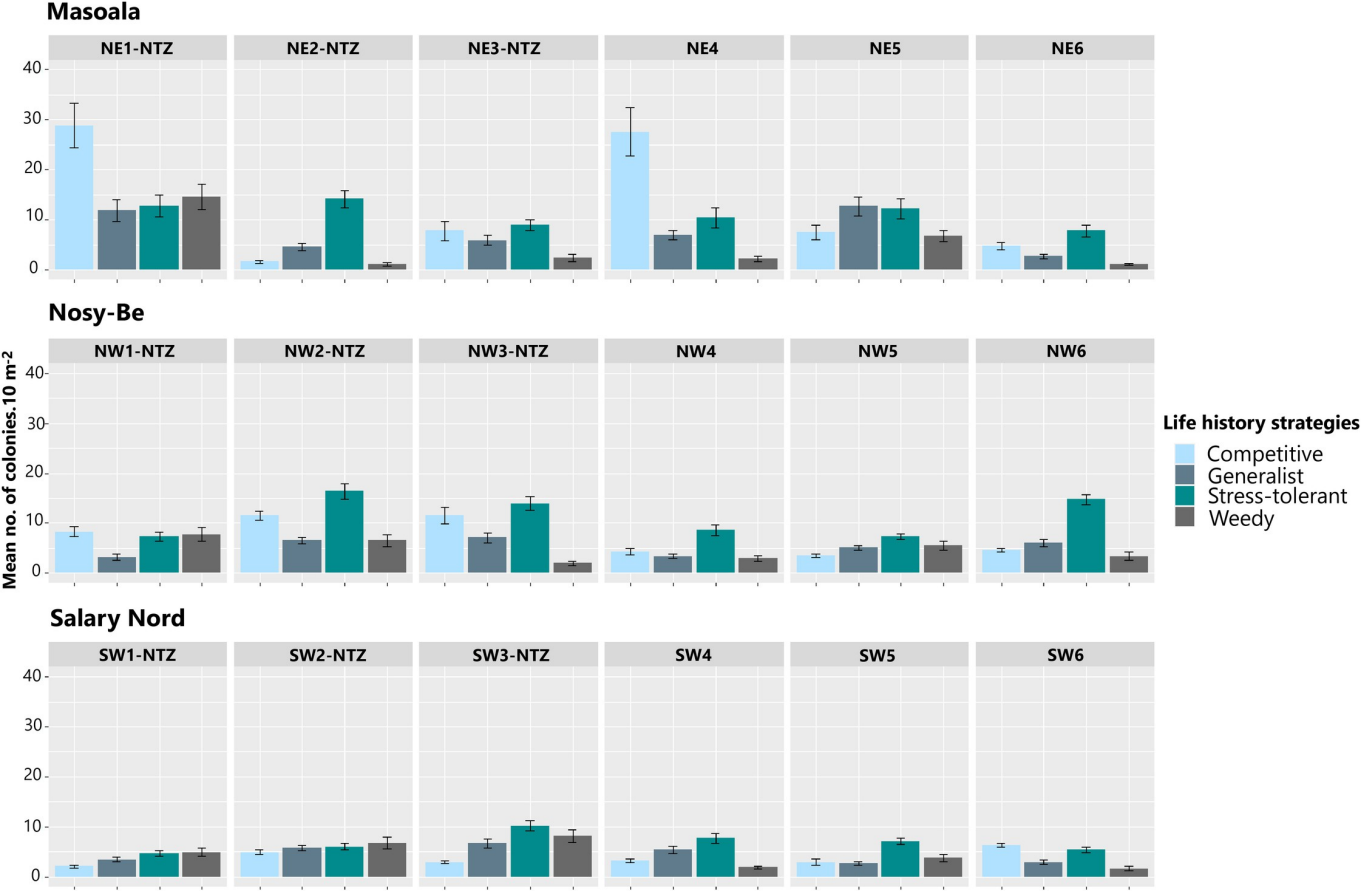
Spatial variation in the abundance of the four coral life history strategies. Data are given for the 18 sampling stations located at the three regions. Errors bars represent standard error.

Coral assemblages were dominated by stress-tolerant taxa at 11 of the 18 stations, whereas competitive taxa were the most represented life history strategy at NE1-NTZ and NE4 in Masoala and, to a lesser degree, at NW1-NTZ in Nosy-Be (Fig 6 and S12 and S13 Tables). Generalist and weedy taxa were generally less abundant except at stations NE5 in Masoala and SW1-NTZ and SW2-NTZ in Salary Nord. Five models were selected as the most parsimonious to explain the variation in the abundance of the four life history strategies (Tables 3 and S14 and S15 and S9 Fig). Region, fishing protection level, life history strategies, herbivorous fish biomass, and the interactions of region × fishing protection level, region × life history strategies, fishing protection level × life history strategies, and region × fishing protection level × life history strategies had the maximum value of relative importance (1.000). Rugosity (0.322), macroalgae cover (0.526), turf (0.169), and CCA (0.169) were also selected by the models, but their relative importance was lower. The models underlined the higher abundance of competitive corals at some Masoala stations, the higher abundance of competitive corals in unfished stations at Nosy-Be, and the higher abundance of weedy corals in unfished stations at Salary Nord.

**Table 3:** Summary of the best linear mixed models using a Poisson distribution to describe variation in abundance of coral life history strategies. FP: fishing protection level, LHS: life history strategies, MA: macroalgae, CCA: crustose coralline algae, HFB: herbivorous fish biomass.

Variables	Intercept	Region	FP	LHS	Rugosity	MA	Turf	CCA	HFB	Region × FP	Region × LHS	FP × LHS	Region × FP × LHS	df	Loglik	AICc	ΔAICc	wAICc	R² marginal
Abundance of life history traits	3.914	×	×	×					0.198	×	×	×	×	26	-1291.27	2642.3	0.00	0.260	0.567
	3.949	×	×	×		- 0.037			0.182	×	×	×	×	27	-1289.99	2642.4	0.10	0.248	0.587
	3.948	×	×	×	0.046				0.198	×	×	×	×	27	-1290.14	2642.7	0.40	0.213	0.577
	3.947	×	×	×		- 0.049	0.022	0.022	0.179	×	×	×	×	28	-1289.04	2643.2	0.87	0.169	0.590
	3.966	×	×	×	0.033	- 0.028			0.186	×	×	×	×	28	-1289.48	2644.0	1.74	0.109	0.578
*Relative importance*		1	1	1	0.322	0.526	0.169	0.169	1	1	1	1	1						
*Coefficient*		Nosy-Be: -0.80 Salary N: -0.89	Unfished: -0.03	Generalist: -0.58 Stress-tol.: -0.26 Weedy: -1.36	0.04	-0.03	0.02	0.18	0.18	Nosy-Be × unfished: 0.86 Salary N × unfished: -0.12	Nosy-Be × generalist: 0.74 Nosy-Be × stress-tol.: 1.17 Nosy-Be × weedy: 1.32 Salary N × generalist: 0.58 Salary N × stress-tol.: 0.84 Salary N × weedy: 0.98	Unfished × generalist: 0.03 Unfished × stress-tol.: 0.20 Unfished × weedy: 0.62	Nosy-Be × unfished × generalist: -0.82 Nosy-Be × unfished × stress-tol.: -0.92 Nosy-Be × unfished × weedy: -1.23 Salary N × unfished × generalist: 0.30 Salary N × unfished × stress-tol.: -0.10 Salary N × unfished × weedy: 0.39						

### Coral cover

The overall coral cover was highly variable within and among regions. Higher cover values were recorded at Nosy-Be (from 42.6% ± 2.9 to 69.8% ± 4.2, mean ± se), compared to Salary Nord (from 25.8% ± 1.3 to 49.7% ± 3.0) and Masoala (from 26.5% ± 0.5 to 46.2% ± 5.6; Fig 4 and S16 Table). The spatial variation in coral cover was supported by seven models (Tables 2 and S17 and S18 and S5 and S10 Figs). Fishing protection level and rugosity had the maximum relative importance (1.000), followed by region (0.603). CCA, turf, and the interaction region × fishing protection level had a lower relative importance (0.474, 0.200 and 0.094, respectively). The models suggested that coral cover increases by 7.04% with an increase of rugosity by 1 unit, and by 8.04% in unfished areas compared to fished ones, this effect being more pronounced at Nosy-Be (12.16% increase; Table 2).

## Discussion

In the context of increasing degradation of coral communities around the globe, as well as the localized threats that Madagascar is facing, our results demonstrate the occurrence of reefs with high diversity, abundance, and cover of corals, including the competitive *Acropora*, which has decreased among other coral reefs. Our study also underlines a positive effect of MPAs on most coral variables, but with varying intensity among regions. These outcomes support Madagascar as a biodiversity hotspot and offer a strong argument for the need to maintain and strengthen conservation and management actions.

### Spatial heterogeneity of coral assemblages

Our survey highlights the marked spatial variability of coral assemblages in Madagascar, with variation at either or both regional and local scales for all coral descriptors. This multiscale spatial heterogeneity is consistent with several previous studies on coral reefs worldwide, and thus represents one of the major characteristics of this ecosystem [6, 9, 57, 93, 94]. As in previous studies in the Western Indian Ocean [6, 38, 57], we found a marked regional-scale variation in the composition of coral assemblages, whereas changes at the local scale (among stations within regions) were less pronounced. Despite this regional dissimilarity in species composition, the local variation of generic richness showed a similar amplitude among regions, with up to two-fold differences among the six stations at each region. This local heterogeneity in generic richness was followed by a similar pattern for the Shannon diversity index, with a particularly high variability at Masoala. Our results also underline a marked heterogeneity at both regional and local scales in the abundance and cover of coral assemblages, with variable contribution of the four life history strategies.

Masoala was characterized by the high abundance of coral colonies (81–345 colonies.10 m^-2^), most notably of the competitive *Acropora* and *Pocillopora as well as* the stress-tolerant taxa *Porites* and *Galaxea* at some stations. The highest abundance values ever recorded at Masoala are greater than the one recorded during this study at Salary Nord, Nosy-Be, or at Toliara on the southwest coast (150–220 colonies.10 m^-2^; [65]). The range of coral cover (~26–46%) that we recorded at Masoala was higher than the one reported by previous studies in this region (~20–35% in 1998, [76]; ~13% in 2005, [68]). Our results support the outcomes of McClanahan [59], who suggested that good ecological conditions of these reefs, with a diverse and abundant coral assemblage and a high dominance of *Acropora* reported in 1996, is probably related to the moderate human impacts in this region. Moreover, the high hydrodynamic and water circulation in the northeast coast [95–98] likely facilitates the larval dispersal of broadcast spawning corals such as *Acropora* and their subsequent regional recruitment. However, this hypothesis should be investigated by a connectivity survey among reefs in this region, which is currently lacking. The good ecological state of the reefs at Masoala seem nevertheless conditioned to the fact that sedimentation and nutrients from rivers do not increase in the coming years [59, 99].

In contrast, Salary Nord was distinguished by lower abundances of coral assemblages, notably with a depauperate population of competitive taxa, mainly *Acropora*. In contrast to the two other regions, a higher proportion of stress-tolerant taxa, such as massive *Porites*, and weedy corals, such as *Seriatopora* and *Stylophora*, were recorded at most stations. The abundance of these taxa, known to colonize and often dominate disturbed environments [5, 100], supports the general consensus that coral reefs of the southwest coast of Madagascar are the most threatened of the island [101]. This lower diversity and abundance of southwestern coral assemblages has been attributed to high-resource extraction and sedimentation [38, 60]. However, the range of coral cover that we recorded at Salary Nord in 2020 (~25–49%) was higher compared to those found by Randriamanantsoa et al. [102] in 2008 (~21–24%). Thus, though comparisons between studies with different sampling methodologies should be taken with caution, our results suggest that Salary Nord reefs have not shown a clear declining trend in recent years.

Coral assemblages at Nosy-Be were characterized by a higher diversity (38 coral genera), higher abundances of several taxa, including *Diploastrea* and *Merulina*, that were much less abundant or not even recorded at the other two regions, and also by the high coral cover (~42–70%) recorded at unfished stations. These high coral cover values, among the highest ever recorded at Madagascar [53, 65], are similar to what was found by Bigot et al. [103] in 1999 (~68% on the outer slope of Nosy Tanihely), but higher than those recorded by Webster & McMahon [66] in the same year (~28–51%). Even if they are confronted by several threats, coral reefs of Nosy-Be are generally considered to be healthier compared to most other reefs around Madagascar, with a high capacity to recover from disturbances [6, 55, 69, 104, 105]. In fact, Nosy-Be is located within the high diversity center in the Northern Mozambique Channel [6, 38, 106, 107]. The high diversity and abundance of coral assemblages in this region is generally associated with the high connectivity among reefs generated by meso-scale eddies [108], and to less frequent storms [6]. This region is therefore considered a priority area for conservation [6].

The present study has focused on the regional and local scales and has provided a valuable contemporary baseline to determine temporal trajectories of coral assemblages, which is crucial for implementing effective management and conservation actions. Given the size of Madagascar and its diversity in environmental conditions, our survey should be reinforced with, for example, the addition of other reef habitats such as shallow back reefs and mesophotic depths, as well as other reefs around the island, notably those from less studied regions such as those in the Kirindy-Mite and Barren Isles in the west, Ambodivahibe in the northeast, and Anosy in the southeast.

### Influence of management status and environmental conditions

Our results clearly underline the positive effects of fishing protection levels on most descriptors of coral assemblages. Stations localized in unfished areas (MPAs) of the three regions showed higher values of generic richness, Shannon diversity index, colony abundance, and cover. The relative contribution of the four life history strategies was also influenced by fishing protection level with, as an example, higher abundance of competitive coral taxa in unfished areas. In fact, species composition was the only component of the coral assemblages that was not related to fishing protection level and rather linked to geographic location. This “MPA effect” was particularly evident at Nosy-Be, with the highest contrast between fished and unfished stations being in terms of abundance and cover of coral colonies, with notably higher abundance of encrusting and massive coral growth forms and reduced macroalgae cover at unfished stations. Size, age, depth and connectivity are major factors in the effectiveness and success of MPAs [31, 32, 36, 46–48]. Here, we suggest that the relatively small size [31, 42, 109] and accessibility of the MPAs are key aspects of the MPA effect at Nosy-Be, facilitating management in terms of monitoring, control, and awareness-raising. Moreover, MPAs at Nosy-Be are the oldest in this study, and this greater time of protection may also explain the highest MPA effect recorded here, consistent with other coral reefs worldwide [46, 47]. Moreover, with several small, scattered islands, and favorable current conditions, the connectivity in the Nosy-Be region is likely a factor that contributes to this positive MPA effect on the higher diversity and abundance of coral assemblages, as recently documented [36, 46, 48].

The biomass of herbivorous fishes was also linked to the spatial variation in generic richness, Shannon diversity index, life history strategies, and abundance of coral assemblages, with higher values of these coral descriptors at stations with high biomass of herbivorous fishes. Herbivory on coral reefs is considered a key function because it often mediates coral-algal interactions in favor of reef-building corals [110, 111]. This expected negative correlation between herbivorous fish biomass and cover of turf and macroalgae was recorded at Masoala and Nosy-Be, but not at Salary Nord. This herbivorous-algal interaction probably explains the negative link that we also recorded between algal cover (turf and macroalgae) and the generic richness, cover, and life history strategies of coral assemblages. These outcomes reinforce the demonstrated mechanism of a high biomass of herbivorous taxa limiting the cover of algae, in turn benefitting coral assemblages, as fleshy algae compete for space and use allelopathy to reduce recruitment, growth, and survivorship of corals [30, 112–115]. This mechanism has been effectively demonstrated in several MPAs [30, 31, 116–118] though could not be confirmed in others [75, 119, 120].

Crustose coralline algae (CCA) cover was positively related to spatial variation of the Shannon diversity index and, to a lesser degree, of generic richness and cover. These relationships could be explained by the fact that some species of CCA induce the metamorphosis and settlement of larvae of several coral species [121–123], and also tend to prevent the establishment and growth of macroalgae by producing antifouling chemical signals [124]. The fact that CCA is a composite metric with not all species inducing coral settlement likely explains the relatively weak, although statistically significant, relationship that we found here, notably for generic richness and cover [125, 126]. Furthermore, the negative link between CCA cover and abundance of coral colonies that we recorded is difficult to interpret. Due to the correlative nature of our analysis, this relationship does not necessarily imply a strong underlying ecological mechanism. Biochemical and habitat-specific cues not considered in this study likely influence the density of coral populations at local scales.

Substrate rugosity was strongly and positively linked with coral cover and composition as well as, at a reduced level, the Shannon diversity index and the relative abundance of the four life history strategies. High rugosity typically provides a larger surface available for sessile organisms as along with more protection from environmental stressors and ecological niche space for marine organisms [127–130]. For several reef invertebrates, including corals, high substrate micro-topography promotes larval settlement and recruitment by reducing mortality by predation up to a point where this factor represents a bottleneck in the replenishment of local populations [122, 131]. Consequently, the reduction of substrate rugosity, notably after storms, cyclones, or gleaning activities, is associated with reef degradation [132–134]. The outcomes of our survey thus reinforce the positive link between rugosity and the diversity and abundance of reef invertebrates and fishes previously recorded [132, 133, 135–137]. However, because the substrate rugosity was visually assessed in the present study, it should be interesting to examine the relationship between rugosity and the structure and dynamics of coral assemblages with the recently developed underwater 3D photogrammetry approach, which allows a much more precise measurement of the structural complexity of reef habitats [130, 138, 139].

Since the interactive effects of multiple biotic and abiotic drivers govern the structure and dynamics of coral assemblages [5–9], it is evident that other environmental factors not selected in the present study could also have a major influence on the spatial patterns revealed here. Sedimentation, light penetration, nutrients dynamics, and temperature regime, all of which have been identified as structuring factors for other coral reefs in the SWIO [57] and around Madagascar [65, 67, 140], should be considered in a future survey.

### Conclusions and perspectives

One of the major results of this study is the identification of reefs with high diversity, abundance, and coverage of corals, including the competitive *Acropora*. These original outcomes are particularly unexpected in the current context of increasing degradation of many coral reefs around the world. However, these optimistic results should be tempered as our survey is only a snapshot in a highly dynamic system and especially as large-scale disturbances, such as thermally induced bleaching events and local stressors associated with human growth, are expected to increase in the near future, with possible shifts in coral assemblage distribution, diversity, and structure [141, 142]. Nevertheless, our survey represents a valuable contemporary baseline to document changes of these reefs and may help to determine if they will provide refuge from future environmental changes.

The positive effects of MPAs on coral assemblages have been clearly demonstrated here. The outcomes of our study strongly support the implementation of MPAs where fishing should be reduced or banned, notably for herbivorous fishes, for which we demonstrated a positive role for the abundance and diversity of coral assemblages, in line with previous studies [28, 30, 42, 143]. Conservation actions should also focus on reducing all fishing activities, such as gleaning and using destructive gears, that strongly alter the reef habitat rugosity and structural complexity that is critical for the health of reef communities [132, 133, 138]. Alternative sources of income, such sea cucumber and seaweed farming, that may reduce the negative effects of overfishing on coral communities, should be also encouraged [140, 144]. The strong spatial variability this study reveals on coral assemblages at the local scale suggests that MPAs should integrate a sufficiently larger scale to capture such heterogeneity [38] but that, in contrast, small reserves are easier to manage in Madagascar given the reduced logistic and human resources generally allotted to them. In fact, priority should be given to the implementation of locally managed marine areas with strong primary user involvement, as has been successfully done for fisheries in the Toliara region [35, 71, 74, 75] and elsewhere, such as in Fiji [42]. The outcomes of our study also confirm the need to maintain and strengthen existing MPAs, as older MPAs are known to be more effective in preventing coral decline [32]. This effort should notably focus on the MPAs of Nosy-Be, as this high diversity area may act as a larval source for other “sink” reefs and may favor their replenishment and resilience [48]. It is also timely to establish long-term interannual monitoring of the reef communities, environmental conditions, and threats at several reefs of various protection levels to precisely examine and improve the effectiveness of coral reef conservation at Madagascar.

## Supporting information

S1 Table Main characteristics of the 18 stations surveyed at the three regions around Madagascar, and major disturbances that were reported in recent decades. (PDF)

S2 Table Composition and abundance (mean number of colonies per 10 m^-2^) of coral assemblages at the 18 stations used for nonmetric multidimensional scaling (nMDS). Standard errors (SE) in brackets.
(PDF)

S3 Table Summary of post-hoc tests to examine differences of coral generic richness between the three regions. (PDF)

S4 Table *t*-test from the linear mixed models to examine differences of coral generic richness between fished and unfished stations. (PDF)

S5 Table Summary of post-hoc tests to examine differences of coral generic richness according to fishing protection level (fished *vs*. unfished areas) at each of the three regions. (PDF)

S6 Table Summary of post-hoc tests to examine differences of coral Shannon diversity index between the three regions. (PDF)

S7 Table *t*-test from the linear mixed models to examine differences of the Shannon diversity index between fished and unfished stations. (PDF)

S8 Table Summary of post-hoc tests to examine differences of the Shannon diversity index according to fishing protection level at each of the three regions. (PDF)

S9 Table Summary of post-hoc tests to examine differences of coral abundance between the three regions. (PDF)

S10 Table *t*-test from the linear mixed models to examine differences of coral abundance between fished and unfished stations. (PDF)

S11 Table Summary of post-hoc tests to examine differences of coral abundance according to fishing protection level at each of the three regions. (PDF)

S12 Table Summary of post-hoc tests to examine differences of abundance of coral life history strategies. (PDF)

S13 Table Summary of post-hoc tests to examine differences of abundance of coral life history strategies between the three regions. (PDF)

S14 Table Summary of post-hoc tests to examine differences of abundance of coral life history strategies according to fishing protection level. (PDF)

S15 Table Summary of post-hoc tests to examine differences of abundance of coral life history strategies according to fishing protection level at each of the three regions. (PDF)

S16 Table Summary of post-hoc tests to examine differences of coral cover between the three regions. (PDF)

S17 Table *t*-test from the linear mixed models to examine differences of coral cover between fished and unfished stations. (PDF)

S18 Table Summary of post-hoc tests to examine differences of coral cover according to fishing protection level at each of the three regions. (PDF)

S19 Table Summary of post-hoc tests to examine differences in macroalgal cover between the three regions. (PDF)

S20 Table Generalized linear mixed models *χ*^*2*^-test to examine differences of macroalgal cover between fished and unfished stations. (PDF)

S21 Table Summary of post-hoc tests to examine differences in turf cover between the three regions. (PDF)

S22 Table Generalized linear mixed models *χ*^*2*^-test to examine differences of turf cover between fished and unfished stations. (PDF)

S23 Table Summary of post-hoc tests to examine differences in crustose coralline algae (CCA) cover between the three regions. (PDF)

S24 Table Generalized linear mixed models *χ*^*2*^-test to examine differences of crustose coralline algae (CCA) cover between fished and unfished stations. (PDF)

S25 Table Summary of post-hoc tests to examine differences in herbivorous fish biomass between the three regions. (PDF)

S26 Table Generalized linear mixed models *χ*^*2*^-test to examine differences of herbivorous fish biomass between fished and unfished stations. (PDF)

S27 Table Summary of post-hoc tests to examine differences in rugosity index between the three regions. (PDF)

S28 Table Generalized linear mixed models *χ*^*2*^-test to examine differences of rugosity index between fished and unfished stations. (PDF)

S1 Fig Correlation analysis between explanatory variables. Spearman correlation coefficients (ρ) were calculated for all stations/regions (overall) and for each of the three regions (above diagonal). Scatterplots of each pair of explanatory variables (below diagonal), and the distribution of variable (diagonal) are also given.
(PDF)

S2 Fig Relationships between coral generic richness and the four explanatory variables (herbivorous fish biomass, fishing protection level, CCA cover, and turf cover) selected by the linear mixed models. Blue lines are linear model fits and red lines are LOESS (locally weighted scatterplot smoothing), with standard error for each (dark grey for linear model fits, and light grey for LOESS). RI: relative importance of each variable.
(PDF)

S3 Fig Relationships between Shannon diversity index and the four explanatory variables (CCA cover, herbivorous fish biomass, fishing protection level, and rugosity) selected by the linear mixed models. Blue lines are linear model fits and red lines are LOESS (locally weighted scatterplot smoothing), with standard error for each (dark grey for linear model fits, and light grey for LOESS). RI: relative importance of each variable.
(PDF)

S4 Fig Relationships between coral abundance and the three explanatory variables (herbivorous fish biomass, fishing protection level, and CCA cover) selected by the linear mixed models. Blue lines are linear model fits and red lines are LOESS (locally weighted scatterplot smoothing), with standard error for each (dark grey for linear model fits, and light grey for LOESS). RI: relative importance of each variable.
(PDF)

S5 Fig Relationships between coral cover and the five explanatory variables (fishing protection level, rugosity, region, CCA cover, and turf cover) selected by the linear mixed models. Blue lines are linear model fits and red lines are LOESS (locally weighted scatterplot smoothing), with standard error for each (dark grey for linear model fits, and light grey for LOESS). RI: relative importance of each variable.
(PDF)

S6 Fig Mean effects of explanatory variables on the spatial variation in coral generic richness. Values have been standardized as effect sizes; circles represent mean parameter estimates and lines represent 95% confidence intervals. Filled dots indicate significant mean values (i.e., different from zero).
(PDF)

S7 Fig Mean effects of explanatory variables on the spatial variation in Shannon diversity index. Values have been standardized as effect sizes; circles represent mean parameter estimates and lines represent 95% confidence intervals. Filled dots indicate significant mean values (i.e., different from zero).
(PDF)

S8 Fig Mean effects of explanatory variables on the spatial variation in coral abundance. Values have been standardized as effect sizes; circles represent mean parameter estimates and lines represent 95% confidence intervals. Filled dots indicate significant mean values (i.e., different from zero).
(PDF)

S9 Fig Mean effects of explanatory variables on the spatial variation in abundance of the four life history strategies. Values have been standardized as effect sizes; circles represent mean parameter estimates, and lines represent 95% confidence intervals. Filled dots indicate significant mean values (i.e., different from zero).
(PDF)

S10 Fig Mean effects of explanatory variables on the spatial variation in coral cover. Values have been standardized as effect sizes, circles represent mean parameter estimates and lines represent 95% confidence intervals. Filled dots indicate significant mean values (i.e., different from zero).
(PDF)
