# Supplementary material for: A contemporary baseline of Madagascar’s coral assemblages: Reefs with high coral diversity, abundance, and function associated with marine protected areas
Source: PLoS One. 2022 Oct 20;17(10):e0275017. doi: 10.1371/journal.pone.0275017 (PMC9584525; doi:10.1371/journal.pone.0275017)
Supplement: S1 Table — (PDF) [file pone.0275017.s001.pdf]

S1 Table. Main characteristics of the 18 stations surveyed at the three regions around Madagascar, and major disturbances reported in recent decades.

Regions	Station name	Station code	Latitude	Longitude	Management status	Fished	Major disturbances
Masoala	Ankoalambano	NE1-NTZ	16°00’54.86”S	50°09’45.60”E	Masoala Marine Park Created in March 1997 Area: 3890 ha	No	Bleaching 1998 [1] Bleaching 2016 [2] Cyclones Hudah 2000, Gafilo 2004, Indlala 2007 [3] Cyclones Enawo 2017, Eliakim 2018
	Ambodilaitry	NE2-NTZ	16°59’41.60”S	50°13’16.84”E		No	
	Nosy Nepato	NE3-NTZ	16°.59’33.74”S	50°13’36.10”E		No	
	Sambo Maty	NE4	16°00’47.09”S	50°09’25.48”E		Yes	
	Ambatomikopaka	NE5	16°00’32.43”S	50°12’14.54”E		Yes	
	Passe Le petit	NE6	16°00’01.87”S	50°12’40.66”E		Yes	
Nosy-Be	Lokobe	NW1-NTZ	13°25’31.68”S	48°19’08.20”E	Lokobe Marine Park Created in September 2011 Area: 122 ha	No	Bleaching 1998 [4] Bleaching 2016 [2] Cyclones Enawo 2017, Belna 2019
	Tanihely sud	NW2-NTZ	13°29’06.73”S	48°13’58.80”E	Nosy Tanihely Protected Area Created in 1966, and modified to a Marine Park in September 2011 Area: 179 ha	No	
	Tanihely ouest	NW3-NTZ	13°28’54.60”S	48°14’00.17”E		No	
	Nosy Tanga	NW4	13°21’56.40”S	48°10’36.80”E	Unprotected	Yes	
	Nosy Komba	NW5	13°26’50.22”S	48°19’52.81”E		Yes	
	Nosy Vorona	NW6	13°25’30.10”S	48°21’42.20”E		Yes	
Salary Nord	Andrefa Mahasaha	SW1-NTZ	22°31’10.38”S	43°14’39.48”E	Soariaka MPA Created in April 2015 Area: 38291 ha	No	Bleaching 1998 [4] Bleaching 2016 [2] Cyclone Haruna 2013 [5]
	Ankaramivony	SW2-NTZ	22°32’44.39”S	43°14’39.02”E		No	
	Anjokozoko	SW3-NTZ	22°37’16.64”S	43°15’27.64”E		No	
	Andravona	SW4	22°29’38.29”S	43°14’23.50”E		Yes	
	Belamera	SW5	22°35’04.80”S	43°15’25.70”E		Yes	
	Tsandamba	SW6	22°44’21.20”S	43°18’58.20”E		Yes	

1. McClanahan TR, Obura D. Monitoring, training and assessment of the coral reefs of the Masoala Peninsula. Project Report to Care-Masoala; 1998.
2. Gudka M, Obura D, Mbugua J, Ahamada S, Kloiber U, Holter T. Participatory reporting of the 2016 bleaching event in the Western Indian Ocean. Coral Reefs. 2020, 39: 1–11.
3. Harding S, Randriamanantsoa B. Coral reef monitoring in marine reserves of northern Madagascar ten years after bleaching–facing the consequences of climate change in the Indian Ocean. CORDIO Status Report; 2008. pp. 93–106.
4. Ahamada S, Bigot L, Bijoux J, Maharavo J, Meunier S, Moyne-Picard M, et al. Status of coral reefs in the south west Indian Ocean Island node: Comoros, Madagascar, Mauritius, Reunion and Seychelles. In: Wilkinson CR, editors. Status of coral reefs of the world. GCRMN Report. Townsville, Australia: Australian Institute of Marine Science; 2002. pp. 79–100
5. Carter AL, Gilchrist H, Dexter KG, Gardner CJ, Gough C, Rocliffe S, Wilson AMW, 2022. Cyclone impacts on coral reef communities in Southwest Madagascar. Frontiers in Marine Science. 2022; 9: 753325.
