# Supplementary material for: A contemporary baseline of Madagascar’s coral assemblages: Reefs with high coral diversity, abundance, and function associated with marine protected areas
Source: PLoS One. 2022 Oct 20;17(10):e0275017. doi: 10.1371/journal.pone.0275017 (PMC9584525; doi:10.1371/journal.pone.0275017)
Supplement: S2 Table — Standard errors (SE) in brackets. (PDF) [file pone.0275017.s002.pdf]

S2 Table. Composition and abundance (mean number of colonies per 10 m^{-2}) of coral assemblages at the 18 stations used for nonmetric multidimensional scaling (nMDS). Standard errors (SE) in brackets.

**Region**	**Masoala**						**Nosy-Be**						**Salary Nord**					
**Station**	**NE1-NTZ**	**NE2-NTZ**	**NE3-NTZ**	**NE4**	**NE5**	**NE6**	**NW1-NTZ**	**NW2-NTZ**	**NW3-NTZ**	**NW4**	**NW5**	**NW6**	**SW1-NTZ**	**SW2-NTZ**	**SW3-NTZ**	**SW4**	**SW5**	**SW6**
Acanthastrea	0.00 (0.00)	2.67 (1.45)	1.00 (0.58)	1.00 (1.00)	2.33 (0.67)	0.00 (0.00)	0.00 (0.00)	3.00 (0.00)	0.00 (0.00)	1.67 (1.67)	0.33 (0.33)	2.00 (2.00)	0.33 (0.33)	0.00 (0.00)	5.00 (3.21)	1.33 (0.88)	0.67 (0.67)	0.00 (0.00)
Acropora	142.33 (19.65)	5.67 (1.67)	38.00 (10.60)	132.33 (2.33)	34.00 (8.54)	22.00 (1.00)	41.00 (3.61)	53.33 (8.69)	51.33 (8.65)	20.00 (2.65)	16.00 (1.15)	22.67 (2.60)	8.67 (1.33)	23.33 (1.45)	12.67 (1.20)	14.33 (2.33)	13.33 (3.48)	30.33 (4.91)
Astreopora	0.00 (0.00)	0.00 (0.00)	0.00 (0.00)	0.00 (0.00)	0.00 (0.00)	0.00 (0.00)	0.00 (0.00)	0.67 (0.67)	0.67 (0.67)	3.33 (1.67)	0.00 (0.00)	0.00 (0.00)	0.00 (0.00)	0.00 (0.00)	0.00 (0.00)	0.00 (0.00)	0.00 (0.00)	0.00 (0.00)
Coscinaraea	0.00 (0.00)	0.00 (0.00)	0.00 (0.00)	0.00 (0.00)	0.00 (0.00)	0.00 (0.00)	0.00 (0.00)	1.33 (1.33)	1.67 (0.88)	0.00 (0.00)	0.00 (0.00)	0.00 (0.00)	0.00 (0.00)	0.00 (0.00)	0.00 (0.00)	0.00 (0.00)	1.00 (1.00)	0.00 (0.00)
Cyphastrea	6.33 (2.03)	0.00 (0.00)	4.33 (2.19)	4.00 (3.06)	2.67 (1.20)	0.00 (0.00)	0.00 (0.00)	3.33 (1.86)	0.00 (0.00)	0.00 (0.00)	0.00 (0.00)	1.67 (1.67)	0.00 (0.00)	0.00 (0.00)	7.33 (2.67)	5.00 (2.08)	0.00 (0.00)	2.00 (1.15)
Diploastrea	0.00 (0.00)	0.00 (0.00)	0.00 (0.00)	0.00 (0.00)	0.00 (0.00)	0.00 (0.00)	0.67 (0.67)	6.00 (1.53)	2.33 (0.67)	1.00 (1.00)	3.33 (0.88)	5.67 (0.33)	0.00 (0.00)	0.00 (0.00)	0.00 (0.00)	0.00 (0.00)	0.00 (0.00)	0.00 (0.00)
Echinopora	0.00 (0.00)	5.67 (1.45)	3.00 (0.58)	2.67 (0.88)	15.67 (4.91)	5.33 (1.20)	8.00 (2.08)	9.33 (1.45)	10.33 (1.76)	7.33 (0.33)	7.67 (2.96)	16.33 (2.96)	2.67 (1.76)	4.67 (0.67)	2.33 (0.88)	0.00 (0.00)	3.00 (1.15)	0.00 (0.00)
Euphyllia	0.00 (0.00)	0.00 (0.00)	0.00 (0.00)	0.00 (0.00)	0.00 (0.00)	0.00 (0.00)	0.00 (0.00)	0.00 (0.00)	0.00 (0.00)	0.00 (0.00)	0.00 (0.00)	0.67 (0.67)	0.00 (0.00)	0.00 (0.00)	0.00 (0.00)	0.00 (0.00)	0.00 (0.00)	0.00 (0.00)
Favia	5.00 (1.53)	7.67 (2.91)	2.33 (0.88)	0.00 (0.00)	4.33 (1.20)	0.00 (0.00)	0.00 (0.00)	4.00 (1.00)	1.67 (0.88)	4.33 (0.33)	1.33 (0.88)	2.33 (0.33)	1.33 (0.33)	3.00 (1.00)	5.33 (0.67)	4.33 (0.33)	0.00 (0.00)	3.33 (0.33)
Favites	18.00 (8.08)	9.67 (1.67)	7.67 (1.20)	5.33 (2.60)	12.67 (3.18)	9.33 (3.18)	0.00 (0.00)	9.67 (5.49)	3.00 (1.53)	8.33 (4.41)	0.00 (0.00)	5.33 (1.33)	1.67 (0.33)	1.33 (0.33)	6.33 (0.88)	7.67 (1.33)	2.00 (1.00)	5.67 (0.33)
Fungia	0.00 (0.00)	4.67 (1.45)	0.00 (0.00)	0.00 (0.00)	0.00 (0.00)	6.00 (2.00)	8.67 (4.91)	5.33 (3.18)	1.67 (1.67)	0.00 (0.00)	6.33 (3.48)	2.67 (1.76)	0.00 (0.00)	3.00 (0.58)	1.67 (0.88)	2.00 (1.00)	2.00 (1.15)	0.00 (0.00)
Galaxea	25.67 (7.84)	15.00 (4.58)	21.67 (4.91)	36.00 (7.51)	18.33 (3.93)	20.33 (2.19)	15.33 (2.73)	12.67 (1.45)	30.33 (7.13)	12.67 (1.45)	9.00 (1.00)	28.67 (8.84)	2.00 (2.00)	1.33 (0.67)	0.00 (0.00)	5.33 (2.33)	1.00 (1.00)	8.67 (1.67)
Gardineroseris	0.00 (0.00)	2.67 (1.20)	2.00 (0.58)	0.00 (0.00)	1.67 (0.67)	0.00 (0.00)	0.00 (0.00)	1.33 (1.33)	1.00 (1.00)	1.00 (1.00)	0.00 (0.00)	2.00 (0.58)	0.00 (0.00)	1.33 (1.33)	0.00 (0.00)	1.67 (0.33)	0.00 (0.00)	0.33 (0.33)
Goniastrea	4.33 (1.20)	1.00 (0.58)	0.00 (0.00)	4.67 (2.60)	0.00 (0.00)	1.00 (0.58)	0.00 (0.00)	0.00 (0.00)	0.00 (0.00)	0.67 (0.67)	1.67 (1.67)	0.00 (0.00)	1.33 (1.33)	0.00 (0.00)	4.33 (1.20)	0.00 (0.00)	0.00 (0.00)	0.00 (0.00)
Goniopora	3.67 (1.67)	3.33 (0.88)	1.67 (1.67)	0.00 (0.00)	3.00 (0.58)	0.00 (0.00)	0.33 (0.33)	2.33 (2.33)	2.67 (1.76)	3.67 (2.73)	0.00 (0.00)	1.67 (1.67)	0.00 (0.00)	1.00 (1.00)	2.33 (1.45)	4.33 (1.86)	4.33 (0.33)	1.00 (0.58)
Herpolitha	0.00 (0.00)	1.00 (0.58)	0.00 (0.00)	0.00 (0.00)	0.00 (0.00)	0.00 (0.00)	1.33 (0.67)	0.00 (0.00)	0.00 (0.00)	0.00 (0.00)	0.33 (0.33)	0.00 (0.00)	0.00 (0.00)	0.00 (0.00)	0.00 (0.00)	0.00 (0.00)	0.00 (0.00)	0.00 (0.00)
Hydnophora	3.33 (1.76)	1.33 (0.67)	0.00 (0.00)	1.00 (1.00)	9.00 (2.52)	0.00 (0.00)	0.00 (0.00)	1.00 (1.00)	0.67 (0.67)	0.67 (0.67)	0.00 (0.00)	0.00 (0.00)	0.00 (0.00)	0.00 (0.00)	0.00 (0.00)	0.00 (0.00)	0.00 (0.00)	0.00 (0.00)
Isopora	0.00 (0.00)	0.00 (0.00)	0.00 (0.00)	0.00 (0.00)	0.00 (0.00)	0.00 (0.00)	0.00 (0.00)	0.00 (0.00)	0.00 (0.00)	0.33 (0.33)	0.00 (0.00)	0.67 (0.67)	0.00 (0.00)	0.00 (0.00)	0.00 (0.00)	0.00 (0.00)	0.00 (0.00)	0.00 (0.00)
Leptastrea	1.67 (0.88)	2.67 (1.20)	1.33 (0.67)	3.00 (3.00)	0.00 (0.00)	0.67 (0.67)	0.00 (0.00)	0.00 (0.00)	0.67 (0.67)	0.00 (0.00)	1.00 (1.00)	0.00 (0.00)	0.00 (0.00)	0.00 (0.00)	0.00 (0.00)	0.00 (0.00)	0.00 (0.00)	0.00 (0.00)
Leptoria	3.67 (1.20)	4.00 (1.00)	1.67 (0.88)	0.00 (0.00)	4.33 (0.67)	1.33 (0.88)	4.33 (0.88)	12.33 (1.20)	5.67 (1.76)	4.33 (1.20)	0.67 (0.67)	2.67 (0.33)	1.33 (0.33)	4.00 (0.58)	5.00 (1.53)	0.00 (0.00)	4.67 (0.33)	1.33 (0.88)
Leptoseris	0.00 (0.00)	0.67 (0.67)	1.67 (0.88)	0.00 (0.00)	2.33 (2.33)	0.00 (0.00)	0.00 (0.00)	1.67 (1.67)	2.00 (2.00)	0.00 (0.00)	0.00 (0.00)	0.67 (0.67)	1.67 (1.67)	3.33 (1.67)	11.67 (5.24)	3.00 (1.73)	6.00 (1.53)	0.00 (0.00)
Lobophyllia	1.00 (0.58)	4.00 (2.08)	0.00 (0.00)	0.00 (0.00)	0.00 (0.00)	0.00 (0.00)	0.00 (0.00)	6.67 (2.19)	3.67 (0.33)	1.33 (0.67)	1.67 (1.20)	1.67 (1.20)	0.67 (0.33)	0.33 (0.33)	2.00 (0.58)	0.00 (0.00)	3.67 (0.88)	0.00 (0.00)
Merulina	0.00 (0.00)	0.33 (0.33)	0.00 (0.00)	0.00 (0.00)	0.00 (0.00)	0.00 (0.00)	2.67 (1.33)	1.33 (1.33)	1.00 (1.00)	1.33 (1.33)	6.00 (3.21)	7.33 (7.33)	0.00 (0.00)	0.00 (0.00)	0.00 (0.00)	0.00 (0.00)	0.00 (0.00)	0.00 (0.00)
Millepora	5.67 (2.19)	1.00 (1.00)	0.00 (0.00)	0.33 (0.33)	0.00 (0.00)	0.00 (0.00)	0.00 (0.00)	0.00 (0.00)	0.00 (0.00)	0.00 (0.00)	0.00 (0.00)	0.00 (0.00)	0.00 (0.00)	1.00 (1.00)	4.67 (1.76)	0.00 (0.00)	0.00 (0.00)	0.00 (0.00)
Montastrea	2.00 (0.58)	1.00 (1.00)	3.00 (1.73)	0.00 (0.00)	3.00 (2.08)	0.00 (0.00)	0.00 (0.00)	1.33 (1.33)	0.67 (0.67)	0.33 (0.33)	1.00 (1.00)	2.33 (1.45)	0.67 (0.67)	0.00 (0.00)	2.33 (0.67)	1.67 (0.67)	0.00 (0.00)	0.33 (0.33)

Montipora	2.33 (1.45)	9.00 (1.53)	3.67 (0.88)	13.33 (4.18)	10.67 (2.73)	6.00 (1.15)	2.00 (2.00)	17.33 (6.77)	27.67 (4.10)	3.67 (1.86)	6.00 (1.53)	1.00 (0.58)	6.33 (0.33)	6.00 (0.58)	8.67 (1.45)	6.33 (0.88)	3.67 (0.88)	5.00 (0.58)
Pachyseris	0.00 (0.00)	0.00 (0.00)	0.00 (0.00)	0.00 (0.00)	0.00 (0.00)	0.00 (0.00)	0.00 (0.00)	0.00 (0.00)	2.00 (1.00)	0.00 (0.00)	7.67 (2.33)	0.00 (0.00)	1.33 (0.67)	0.00 (0.00)	2.33 (0.33)	4.00 (1.53)	0.00 (0.00)	0.00 (0.00)
Pavona	26.33 (8.29)	3.33 (0.88)	3.00 (1.53)	10.00 (3.21)	4.33 (1.33)	0.00 (0.00)	0.00 (0.00)	3.67 (3.67)	3.33 (1.76)	0.00 (0.00)	0.00 (0.00)	2.00 (2.00)	5.33 (5.33)	5.67 (3.84)	7.33 (0.88)	0.00 (0.00)	3.67 (2.33)	0.00 (0.00)
Physogyra	0.00 (0.00)	0.00 (0.00)	0.00 (0.00)	0.00 (0.00)	0.00 (0.00)	0.00 (0.00)	0.00 (0.00)	0.67 (0.67)	0.00 (0.00)	0.00 (0.00)	0.33 (0.33)	0.00 (0.00)	0.00 (0.00)	0.33 (0.33)	0.00 (0.00)	0.00 (0.00)	0.00 (0.00)	0.00 (0.00)
Platygyra	0.00 (0.00)	4.33 (0.33)	0.00 (0.00)	0.00 (0.00)	7.33 (1.76)	0.00 (0.00)	0.00 (0.00)	2.00 (1.15)	2.67 (0.33)	0.00 (0.00)	4.00 (1.00)	2.00 (1.15)	2.00 (1.00)	0.33 (0.33)	0.00 (0.00)	3.33 (1.86)	0.00 (0.00)	4.33 (0.67)
Pleurogyra	0.00 (0.00)	0.00 (0.00)	0.00 (0.00)	0.00 (0.00)	0.00 (0.00)	0.00 (0.00)	0.00 (0.00)	2.67 (1.33)	1.00 (1.00)	0.00 (0.00)	0.33 (0.33)	0.33 (0.33)	0.00 (0.00)	0.33 (0.33)	0.67 (0.33)	0.00 (0.00)	0.67 (0.67)	0.00 (0.00)
Pocillopora	29.33 (3.76)	6.67 (2.96)	13.00 (2.65)	10.33 (2.19)	35.33 (7.51)	5.67 (2.33)	5.67 (5.17)	0.00 (0.00)	2.00 (0.58)	8.33 (2.03)	0.00 (0.00)	3.67 (1.33)	6.33 (1.33)	19.67 (2.33)	12.00 (1.15)	17.00 (2.65)	3.00 (1.53)	13.33 (2.19)
Podabacia	0.00 (0.00)	0.00 (0.00)	0.00 (0.00)	0.00 (0.00)	0.00 (0.00)	0.00 (0.00)	0.33 (0.33)	0.00 (0.00)	0.00 (0.00)	0.00 (0.00)	0.00 (0.00)	0.00 (0.00)	0.00 (0.00)	0.00 (0.00)	0.00 (0.00)	0.00 (0.00)	0.00 (0.00)	0.00 (0.00)
Porites	43.00 (2.89)	6.33 (1.33)	6.00 (1.15)	8.00 (0.58)	9.33 (1.76)	0.00 (0.00)	26.00 (10.21)	16.00 (1.53)	10.67 (3.53)	6.33 (3.18)	9.00 (4.04)	28.33 (9.39)	9.67 (1.76)	23.33 (4.98)	16.00 (5.77)	3.00 (0.00)	9.00 (1.15)	1.00 (1.00)
Psammocora	0.00 (0.00)	0.00 (0.00)	0.00 (0.00)	1.00 (1.00)	0.00 (0.00)	0.00 (0.00)	0.00 (0.00)	4.33 (2.33)	0.00 (0.00)	0.00 (0.00)	0.00 (0.00)	0.00 (0.00)	0.00 (0.00)	0.00 (0.00)	0.00 (0.00)	2.33 (1.20)	2.33 (0.67)	0.00 (0.00)
Seriatopora	16.67 (6.17)	3.33 (0.33)	2.67 (1.45)	0.00 (0.00)	9.67 (5.93)	1.67 (1.20)	14.67 (5.81)	21.67 (3.18)	2.67 (1.20)	6.00 (3.21)	23.67 (1.45)	0.00 (0.00)	13.33 (1.86)	10.33 (1.33)	15.00 (3.21)	4.00 (1.00)	15.67 (0.67)	4.00 (0.58)
Stylophora	5.33 (2.40)	0.00 (0.00)	1.00 (1.00)	3.00 (1.53)	6.33 (2.19)	2.33 (1.45)	1.67 (0.88)	0.00 (0.00)	0.33 (0.33)	0.00 (0.00)	0.00 (0.00)	0.00 (0.00)	8.33 (1.45)	4.33 (0.88)	9.33 (1.76)	0.00 (0.00)	2.33 (0.33)	0.67 (0.33)
Symphyllia	0.00 (0.00)	1.00 (0.58)	0.00 (0.00)	0.00 (0.00)	0.00 (0.00)	0.00 (0.00)	0.00 (0.00)	0.33 (0.33)	0.00 (0.00)	0.00 (0.00)	0.33 (0.33)	0.00 (0.00)	0.00 (0.00)	0.00 (0.00)	0.00 (0.00)	0.00 (0.00)	0.00 (0.00)	0.00 (0.00)
Tubastrea	0.00 (0.00)	0.00 (0.00)	0.00 (0.00)	0.00 (0.00)	0.00 (0.00)	0.00 (0.00)	0.00 (0.00)	0.00 (0.00)	0.00 (0.00)	0.00 (0.00)	0.67 (0.67)	0.00 (0.00)	0.00 (0.00)	0.00 (0.00)	0.00 (0.00)	0.00 (0.00)	0.00 (0.00)	0.00 (0.00)
Turbinaria	0.00 (0.00)	0.00 (0.00)	6.33 (2.60)	0.00 (0.00)	0.00 (0.00)	0.00 (0.00)	0.00 (0.00)	0.00 (0.00)	0.00 (0.00)	0.00 (0.00)	0.00 (0.00)	0.00 (0.00)	0.00 (0.00)	0.00 (0.00)	0.00 (0.00)	0.67 (0.67)	0.67 (0.67)	0.00 (0.00)
