# Supplementary material for: A contemporary baseline of Madagascar’s coral assemblages: Reefs with high coral diversity, abundance, and function associated with marine protected areas
Source: PLoS One. 2022 Oct 20;17(10):e0275017. doi: 10.1371/journal.pone.0275017 (PMC9584525; doi:10.1371/journal.pone.0275017)
Supplement: S4 Table — (PDF) [file pone.0275017.s004.pdf]

S4 Table. *t*-test from the linear mixed models to examine differences of coral generic richness between fished and unfished stations. Significant *P*-values (<0.05) are highlighted in bold (*: <0.05, **: <0.01, ***: <0.001).

**Contrast**		**Estimate**	**SE**	**df**	***t*.value**	***P*-value**	
Unfished	Fished	5.15	2.17	11.83	2.37	**0.0354**	*
