# Supplementary material for: A contemporary baseline of Madagascar’s coral assemblages: Reefs with high coral diversity, abundance, and function associated with marine protected areas
Source: PLoS One. 2022 Oct 20;17(10):e0275017. doi: 10.1371/journal.pone.0275017 (PMC9584525; doi:10.1371/journal.pone.0275017)
Supplement: S5 Table — (PDF) [file pone.0275017.s005.pdf]

S5 Table. Summary of post-hoc tests to examine differences of coral generic richness according to fishing protection level (fished *vs.* unfished areas) at each of the three regions. Significant *P*-values (<0.05) are highlighted in bold (*: <0.05, **: <0.01, ***: <0.001).

**Contrast**		**Estimate**	**SE**	**df**	***t*.ratio**	***P*-value**
Masoala						
Fished	Unfished	-5.15	2.96	40.30	-1.73	0.0897
Nosy-Be						
Fished	Unfished	-0.87	3.11	47.00	-0.28	0.7804
Salary Nord						
Fished	Unfished	-1.38	3.02	41.80	-0.45	0.6500
