# Supplementary material for: A contemporary baseline of Madagascar’s coral assemblages: Reefs with high coral diversity, abundance, and function associated with marine protected areas
Source: PLoS One. 2022 Oct 20;17(10):e0275017. doi: 10.1371/journal.pone.0275017 (PMC9584525; doi:10.1371/journal.pone.0275017)
Supplement: S6 Table — (PDF) [file pone.0275017.s006.pdf]

S6 Table. Summary of post-hoc tests to examine differences of coral Shannon diversity index between the three regions. Significant *P*-values (<0.05) are highlighted in bold (*: <0.05, **: <0.01, ***: <0.001).

**Contrast**		**Estimate**	**SE**	**df**	***t*.ratio**	***P*-value**
Masoala	Nosy-Be	-0.07	0.19	21.50	-0.37	0.9255
Masoala	Salary Nord	-0.18	0.19	21.80	-0.95	0.6138
Nosy-Be	Salary Nord	-0.11	0.19	21.80	-0.57	0.8329
