# Supplementary material for: A contemporary baseline of Madagascar’s coral assemblages: Reefs with high coral diversity, abundance, and function associated with marine protected areas
Source: PLoS One. 2022 Oct 20;17(10):e0275017. doi: 10.1371/journal.pone.0275017 (PMC9584525; doi:10.1371/journal.pone.0275017)
Supplement: S7 Table — (PDF) [file pone.0275017.s007.pdf]

S7 Table. *t*-test from the linear mixed models to examine differences of the Shannon diversity index between fished and unfished stations. Significant *P*-values (<0.05) are highlighted in bold (*: <0.05, **: <0.01, ***: <0.001).

Contrast		Estimate	SE	df	*t*.value	*P*-value
Unfished	Fished	0.40	0.20	13.30	1.98	0.0677
