# Supplementary material for: A contemporary baseline of Madagascar’s coral assemblages: Reefs with high coral diversity, abundance, and function associated with marine protected areas
Source: PLoS One. 2022 Oct 20;17(10):e0275017. doi: 10.1371/journal.pone.0275017 (PMC9584525; doi:10.1371/journal.pone.0275017)
Supplement: S8 Table — (PDF) [file pone.0275017.s008.pdf]

S8 Table. Summary of post-hoc tests to examine differences of the Shannon diversity index according to fishing protection level at each of the three regions. Significant *P*-values (<0.05) are highlighted in bold (*: <0.05, **: <0.01, ***: <0.001).

**Contrast**		**Estimate**	**SE**	**df**	***t*.ratio**	***P*-value**
Masoala						
Fished	Unfished	-0.40	0.27	36.80	-1.49	0.1444
Nosy-Be						
Fished	Unfished	0.10	0.28	42.50	0.37	0.1797
Salary Nord						
Fished	Unfished	-0.10	0.27	38.20	-0.37	0.7086
