# Supplementary material for: A contemporary baseline of Madagascar’s coral assemblages: Reefs with high coral diversity, abundance, and function associated with marine protected areas
Source: PLoS One. 2022 Oct 20;17(10):e0275017. doi: 10.1371/journal.pone.0275017 (PMC9584525; doi:10.1371/journal.pone.0275017)
Supplement: S9 Table — (PDF) [file pone.0275017.s009.pdf]

S9 Table. Summary of post-hoc tests to examine differences of coral abundance between the three regions. Significant *P*-values (<0.05) are highlighted in bold (*: <0.05, **: <0.01, ***: <0.001).

**Contrast**		**Estimate**	**SE**	**df**	***t*.ratio**	***P*-value**	
Masoala	Nosy-Be	4.82	2.92	21.70	1.65	0.2468	
Masoala	Salary Nord	7.48	2.93	22.00	2.55	**0.0460**	*
Nosy-Be	Salary Nord	2.66	2.92	21.70	0.91	0.6384	
