# Supplementary material for: A contemporary baseline of Madagascar’s coral assemblages: Reefs with high coral diversity, abundance, and function associated with marine protected areas
Source: PLoS One. 2022 Oct 20;17(10):e0275017. doi: 10.1371/journal.pone.0275017 (PMC9584525; doi:10.1371/journal.pone.0275017)
Supplement: S11 Table — (PDF) [file pone.0275017.s011.pdf]

S11 Table. Summary of post-hoc tests to examine differences of coral abundance according to fishing protection level at each of the three regions. Significant *P*-values (<0.05) are highlighted in bold (*: <0.05, **: <0.01, ***: <0.001).

**Contrast**		**Estimate**	**SE**	**df**	***t*.ratio**	***P*-value**
Masoala						
Fished	Unfished	-21.80	62.40	32.90	-0.34	0.7294
Nosy-Be						
Fished	Unfished	-31.40	63.60	35.90	-0.49	0.6249
Salary Nord						
Fished	Unfished	-23.60	62.80	33.70	-0.37	0.7100
