# Supplementary material for: A contemporary baseline of Madagascar’s coral assemblages: Reefs with high coral diversity, abundance, and function associated with marine protected areas
Source: PLoS One. 2022 Oct 20;17(10):e0275017. doi: 10.1371/journal.pone.0275017 (PMC9584525; doi:10.1371/journal.pone.0275017)
Supplement: S12 Table — (PDF) [file pone.0275017.s012.pdf]

S12 Table. Summary of post-hoc tests to examine differences of abundance of coral life history strategies. Significant *P*-values (<0.05) are highlighted in bold (*: <0.05, **: <0.01, ***: <0.001).

**Contrast**		**Estimate**	**SE**	**df**	***z*.ratio**	***P*-value**	
Competitive	Generalist	0.35	0.03	193.00	10.52	**0.0217**	*
Competitive	Stress-tolerant	-0.18	0.02	193.00	-6.38	0.1939	
Competitive	Weedy	0.56	0.03	193.00	15.64	**<0.0003**	***
Generalist	Stress-tolerant	-0.54	0.03	193.00	-16.65	**<0.0001**	***
Generalist	Weedy	0.20	0.03	193.00	5.36	0.5813	
Stress-tolerant	Weedy	0.75	0.03	193.00	21.51	**<0.0001**	***
