# Supplementary material for: A contemporary baseline of Madagascar’s coral assemblages: Reefs with high coral diversity, abundance, and function associated with marine protected areas
Source: PLoS One. 2022 Oct 20;17(10):e0275017. doi: 10.1371/journal.pone.0275017 (PMC9584525; doi:10.1371/journal.pone.0275017)
Supplement: S13 Table — (PDF) [file pone.0275017.s013.pdf]

S13 Table. Summary of post-hoc tests to examine differences of abundance of coral life history strategies between the three regions. Significant *P*-values (<0.05) are highlighted in bold (*: <0.05, **: <0.01, ***: <0.001).

**Contrast**		**Estimate**	**SE**	**df**	***z*.ratio**	***P*-value**	
Competitive							
Masoala	Nosy-Be	0.49	0.24	45.60	2.41	**0.0416**	*
Masoala	Salary Nord	1.17	0.20	47.80	5.63	**<0.0001**	***
Nosy-Be	Salary Nord	0.68	0.21	47.80	3.25	**0.0033**	**
Generalist							
Masoala	Nosy-Be	0.26	0.20	45.60	1.26	0.4157	
Masoala	Salary Nord	0.43	0.20	47.80	2.06	0.0969	
Nosy-Be	Salary Nord	0.16	0.21	47.80	0.80	0.6976	
Stress-tolerant							
Masoala	Nosy-Be	-0.11	0.20	45.60	-0.55	0.8418	
Masoala	Salary Nord	0.38	0.20	47.80	1.89	0.1409	
Nosy-Be	Salary Nord	0.50	0.20	47.80	2.44	**0.0382**	**
Weedy							
Masoala	Nosy-Be	-0.07	0.20	45.60	-0.37	0.6712	
Masoala	Salary Nord	-0.06	0.21	47.80	0.30	1.0000	
Nosy-Be	Salary Nord	0.01	0.21	47.80	0.06	0.6642	
