# Supplementary material for: A contemporary baseline of Madagascar’s coral assemblages: Reefs with high coral diversity, abundance, and function associated with marine protected areas
Source: PLoS One. 2022 Oct 20;17(10):e0275017. doi: 10.1371/journal.pone.0275017 (PMC9584525; doi:10.1371/journal.pone.0275017)
Supplement: S14 Table — (PDF) [file pone.0275017.s014.pdf]

S14 Table. Summary of post-hoc tests to examine differences of abundance of coral life history strategies according to fishing protection level. Significant *P*-values (<0.05) are highlighted in bold (*: <0.05, **: <0.01, ***: <0.001).

**Contrast**		**Estimate**	**SE**	**df**	***z*.ratio**	***P*-value**	
Competitive							
Fished	Unfished	-0.22	0.18	41.50	-1.17	0.2384	
Generalist							
Fished	Unfished	-0.08	0.18	41.50	-0.46	0.6427	
Stress-tolerant							
Fished	Unfished	-0.07	0.18	41.50	-0.42	0.6708	
Weedy							
Fished	Unfished	-0.56	0.19	41.50	-2.96	**0.0030**	**
