# Supplementary material for: A contemporary baseline of Madagascar’s coral assemblages: Reefs with high coral diversity, abundance, and function associated with marine protected areas
Source: PLoS One. 2022 Oct 20;17(10):e0275017. doi: 10.1371/journal.pone.0275017 (PMC9584525; doi:10.1371/journal.pone.0275017)
Supplement: S15 Table — (PDF) [file pone.0275017.s015.pdf]

S15 Table. Summary of post-hoc tests to examine differences of abundance of coral life history strategies according to fishing protection level at each of the three regions. Significant *P*-values (<0.05) are highlighted in bold (*: <0.05, **: <0.01, ***: <0.001).

Contrast		Estimate	SE	df	*z*.ratio	*P*-value	
Competitive in Masoala							
Fished	Unfished	0.04	0.30	58.50	0.15	0.8733	
Competitive in Nosy-Be							
Fished	Unfished	-0.75	0.31	58.50	-2.38	**0.0172**	*
Competitive in Salary Nord							
Fished	Unfished	0.13	0.32	63.30	0.42	0.6740	
Generalist in Masoala							
Fished	Unfished	0.01	0.32	58.50	0.03	0.9712	
Generalist in Nosy-Be							
Fished	Unfished	0.03	0.32	58.50	0.09	0.9253	
Generalist in Salary Nord							
Fished	Unfished	-0.21	0.31	63.30	-0.65	0.5134	
Stress-tolerant in Masoala							
Fished	Unfished	-0.15	0.31	58.50	-0.49	0.6220	
Stress-tolerant in Nosy-Be							
Fished	Unfished	-0.03	0.31	58.50	-0.10	0.9188	
Stress-tolerant in Salary Nord							
Fished	Unfished	0.04	0.31	63.30	0.13	0.8956	
Weedy in Masoala							
Fished	Unfished	-0.57	0.32	58.50	-1.79	0.0732	
Weedy in Nosy-Be							
Fished	Unfished	-0.14	0.32	58.50	-0.45	0.6508	
Weedy in Salary Nord							
Fished	Unfished	-0.88	0.32	63.30	-2.71	**0.0067**	**
