# Supplementary material for: A contemporary baseline of Madagascar’s coral assemblages: Reefs with high coral diversity, abundance, and function associated with marine protected areas
Source: PLoS One. 2022 Oct 20;17(10):e0275017. doi: 10.1371/journal.pone.0275017 (PMC9584525; doi:10.1371/journal.pone.0275017)
Supplement: S16 Table — (PDF) [file pone.0275017.s016.pdf]

S16 Table. Summary of post-hoc tests to examine differences of coral cover between the three regions. Significant *P*-values (<0.05) are highlighted in bold (*: <0.05, **: <0.01, ***: <0.001).

Contrast		Estimate	SE	df	*t*.ratio	*P*-value	
Masoala	Nosy-Be	-17.30	5.23	21.40	-3.30	**0.0088**	**
Masoala	Salary Nord	-3.79	5.25	21.60	-0.72	0.7535	
Nosy-Be	Salary Nord	13.51	5.25	21.60	2.57	**0.0441**	*
