# Supplementary material for: A contemporary baseline of Madagascar’s coral assemblages: Reefs with high coral diversity, abundance, and function associated with marine protected areas
Source: PLoS One. 2022 Oct 20;17(10):e0275017. doi: 10.1371/journal.pone.0275017 (PMC9584525; doi:10.1371/journal.pone.0275017)
Supplement: S18 Table — (PDF) [file pone.0275017.s018.pdf]

S18 Table. Summary of post-hoc tests to examine differences of coral cover according to fishing protection level at each of the three regions. Significant *P*-values (<0.05) are highlighted in bold (*: <0.05, **: <0.01, ***: <0.001).

Contrast		Estimate	SE	df	*t*.ratio	*P*-value	
Masoala							
Fished	Unfished	-2.40	5.26	35.40	-0.45	0.6509	
Nosy-Be							
Fished	Unfished	-14.7	5.51	41.10	-2.66	**0.0110**	**
Salary Nord							
Fished	Unfished	-9.60	5.36	36.90	-1.79	0.0816	
