# Supplementary material for: A contemporary baseline of Madagascar’s coral assemblages: Reefs with high coral diversity, abundance, and function associated with marine protected areas
Source: PLoS One. 2022 Oct 20;17(10):e0275017. doi: 10.1371/journal.pone.0275017 (PMC9584525; doi:10.1371/journal.pone.0275017)
Supplement: S19 Table — (PDF) [file pone.0275017.s019.pdf]

S19 Table. Summary of post-hoc tests to examine differences in macroalgal cover between the three regions. Significant *P*-values (<0.05) are highlighted in bold (*: <0.05, **: <0.01, ***: <0.001).

Contrast		Estimate	SE	df	*z*.ratio	*P*-value
Masoala	Nosy-Be	0.32	0.34	22.6	0.93	0.6188
Masoala	Salary Nord	0.08	0.34	23.2	0.25	0.9662
Nosy-Be	Salary Nord	-0.23	0.35	23.2	-0.67	0.7760
