# Supplementary material for: A contemporary baseline of Madagascar’s coral assemblages: Reefs with high coral diversity, abundance, and function associated with marine protected areas
Source: PLoS One. 2022 Oct 20;17(10):e0275017. doi: 10.1371/journal.pone.0275017 (PMC9584525; doi:10.1371/journal.pone.0275017)
Supplement: S21 Table — (PDF) [file pone.0275017.s021.pdf]

S21 Table. Summary of post-hoc tests to examine differences in turf cover between the three regions. Significant *P*-values (<0.05) are highlighted in bold (*: <0.05, **: <0.01, ***: <0.001).

**Contrast**		**Estimate**	**SE**	**df**	***z*.ratio**	***P*-value**
Masoala	Nosy-Be	0.10	0.14	22.2	0.76	0.7274
Masoala	Salary Nord	0.09	0.13	23.5	0.71	0.7561
Nosy-Be	Salary Nord	-0.06	0.14	23.5	-0.04	0.9987
