# Supplementary material for: A contemporary baseline of Madagascar’s coral assemblages: Reefs with high coral diversity, abundance, and function associated with marine protected areas
Source: PLoS One. 2022 Oct 20;17(10):e0275017. doi: 10.1371/journal.pone.0275017 (PMC9584525; doi:10.1371/journal.pone.0275017)
Supplement: S22 Table — (PDF) [file pone.0275017.s022.pdf]

S22 Table. Generalized linear mixed models χ^2-test to examine differences of turf cover between fished and unfished stations. Significant *P*-values (<0.05) are highlighted in bold (*: <0.05, **: <0.01, ***: <0.001).

**Contrast**		**Estimate**	**SE**	***z*.value**	***P*-value**
Unfished	Fished	-0.09	0.11	-0.83	0.4040
