# Supplementary material for: A contemporary baseline of Madagascar’s coral assemblages: Reefs with high coral diversity, abundance, and function associated with marine protected areas
Source: PLoS One. 2022 Oct 20;17(10):e0275017. doi: 10.1371/journal.pone.0275017 (PMC9584525; doi:10.1371/journal.pone.0275017)
Supplement: S23 Table — (PDF) [file pone.0275017.s023.pdf]

S23 Table. Summary of post-hoc tests to examine differences in crustose coralline algae (CCA) cover between the three regions. Significant *P*-values (<0.05) are highlighted in bold (*: <0.05, **: <0.01, ***: <0.001).

**Contrast**		**Estimate**	**SE**	**df**	***z*.ratio**	***P*-value**
Masoala	Nosy-Be	0.06	0.39	24.1	0.15	0.9869
Masoala	Salary Nord	-0.62	0.36	25.2	-1.72	0.1959
Nosy-Be	Salary Nord	-0.69	0.36	25.2	-1.87	0.1470
