# Supplementary material for: A contemporary baseline of Madagascar’s coral assemblages: Reefs with high coral diversity, abundance, and function associated with marine protected areas
Source: PLoS One. 2022 Oct 20;17(10):e0275017. doi: 10.1371/journal.pone.0275017 (PMC9584525; doi:10.1371/journal.pone.0275017)
Supplement: S24 Table — (PDF) [file pone.0275017.s024.pdf]

S24 Table. Generalized linear mixed models χ^2-test to examine differences of crustose coralline algae (CCA) cover between fished and unfished stations. Significant *P*-values (<0.05) are highlighted in bold (*: <0.05, **: <0.01, ***: <0.001).

Contrast		Estimate	SE	*z*.value	*P*-value
Unfished	Fished	-0.32	0.30	-1.09	0.2816
