# Supplementary material for: A contemporary baseline of Madagascar’s coral assemblages: Reefs with high coral diversity, abundance, and function associated with marine protected areas
Source: PLoS One. 2022 Oct 20;17(10):e0275017. doi: 10.1371/journal.pone.0275017 (PMC9584525; doi:10.1371/journal.pone.0275017)
Supplement: S25 Table — (PDF) [file pone.0275017.s025.pdf]

S25 Table. Summary of post-hoc tests to examine differences in herbivorous fish biomass between the three regions. Significant *P*-values (<0.05) are highlighted in bold (*: <0.05, **: <0.01, ***: <0.001).

Contrast		Estimate	SE	df	*z*.ratio	*P*-value	
Masoala	Nosy-Be	1.71	0.55	22.8	3.08	**0.0058**	**
Masoala	Salary Nord	1.22	0.55	23.1	2.19	0.0722	
Nosy-Be	Salary Nord	-0.49	0.55	23.1	-0.88	0.6488	
