# Supplementary material for: A contemporary baseline of Madagascar’s coral assemblages: Reefs with high coral diversity, abundance, and function associated with marine protected areas
Source: PLoS One. 2022 Oct 20;17(10):e0275017. doi: 10.1371/journal.pone.0275017 (PMC9584525; doi:10.1371/journal.pone.0275017)
Supplement: S27 Table — (PDF) [file pone.0275017.s027.pdf]

S27 Table. Summary of post-hoc tests to examine differences in rugosity index between the three regions. Significant *P*-values (<0.05) are highlighted in bold (*: <0.05, **: <0.01, ***: <0.001).

**Contrast**		**Estimate**	**SE**	**df**	***z*.ratio**	***P*-value**	
Masoala	Nosy-Be	-0.47	0.17	23.1	-2.66	**0.0211**	*
Masoala	Salary Nord	-0.18	0.18	23.3	-0.97	0.5948	
Nosy-Be	Salary Nord	0.29	0.18	23.3	1.63	0.2313	
