# Supplementary material for: A contemporary baseline of Madagascar’s coral assemblages: Reefs with high coral diversity, abundance, and function associated with marine protected areas
Source: PLoS One. 2022 Oct 20;17(10):e0275017. doi: 10.1371/journal.pone.0275017 (PMC9584525; doi:10.1371/journal.pone.0275017)
Supplement: S1 Fig — Spearman correlation coefficients (ρ) were calculated for all stations/regions (overall) and for each of the three regions (above diagonal). Scatterplots of each pair of explanatory variables (below diagonal), and the distribution of variable (diagonal) are also given. (PDF) [file pone.0275017.s029.pdf]

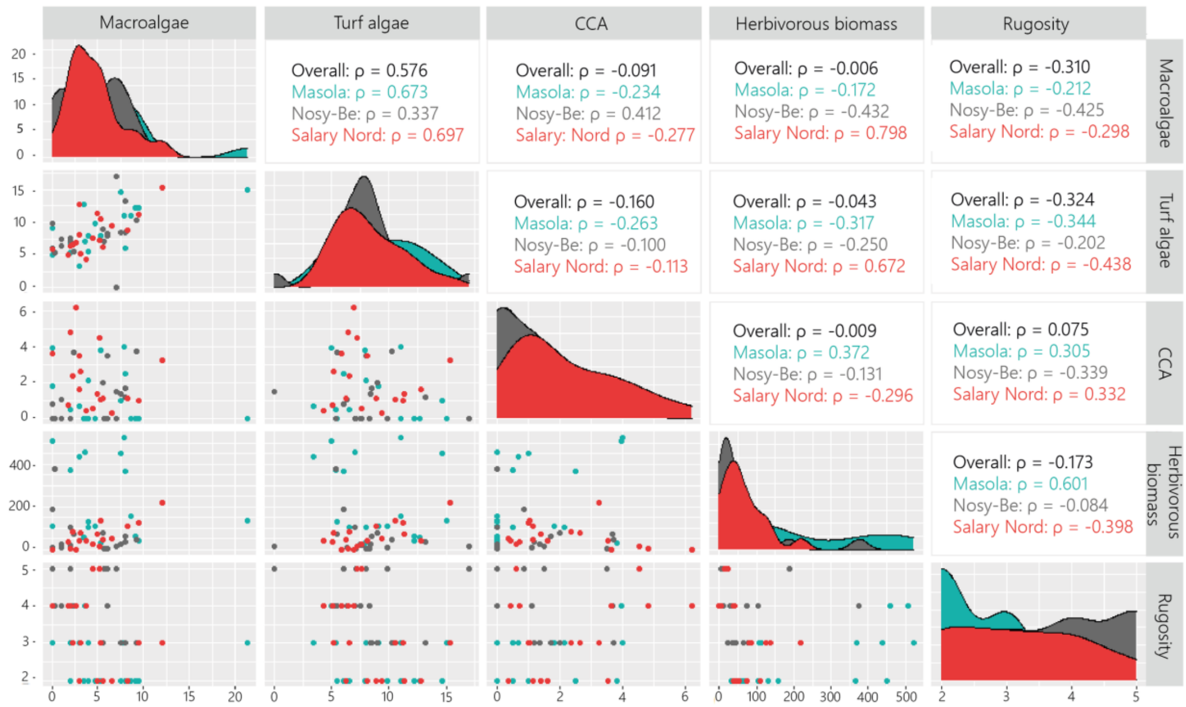

S1 Fig. Correlation analysis between explanatory variables. Spearman correlation coefficients (ρ) were calculated for all stations/regions (overall) and for each of the three regions (above diagonal). Scatterplots of each pair of explanatory variables (below diagonal), and the distribution of variable (diagonal) are also given.
