# Supplementary material for: A contemporary baseline of Madagascar’s coral assemblages: Reefs with high coral diversity, abundance, and function associated with marine protected areas
Source: PLoS One. 2022 Oct 20;17(10):e0275017. doi: 10.1371/journal.pone.0275017 (PMC9584525; doi:10.1371/journal.pone.0275017)
Supplement: S4 Fig — Blue lines are linear model fits and red lines are LOESS (locally weighted scatterplot smoothing), with standard error for each (dark grey for linear model fits, and light grey for LOESS). RI: relative importance of each variable. (PDF) [file pone.0275017.s032.pdf]

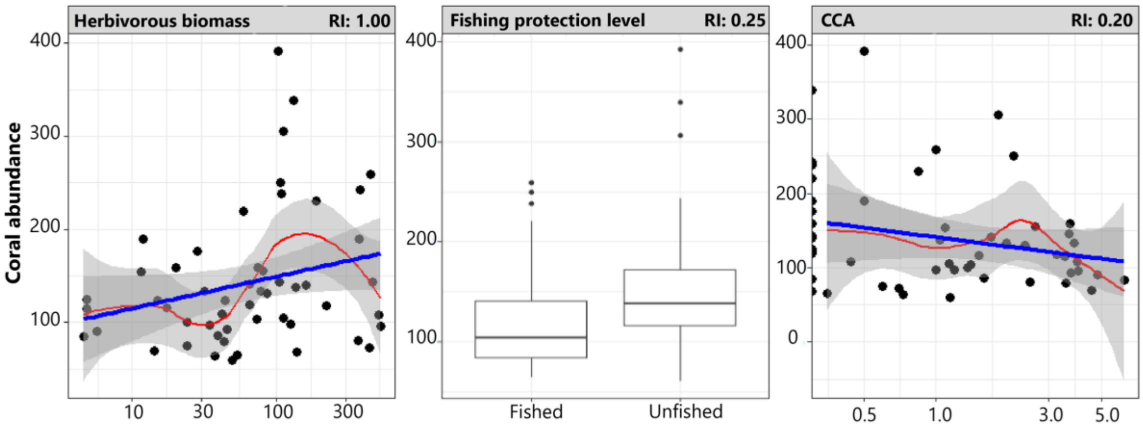

S4 Fig. Relationships between coral abundance and the three explanatory variables (herbivorous fish biomass, fishing protection level, and CCA cover) selected by the linear mixed models. Blue lines are linear model fits and red lines are LOESS (locally weighted scatterplot smoothing), with standard error for each (dark grey for linear model fits, and light grey for LOESS). RI: relative importance of each variable.
