# Supplementary material for: A contemporary baseline of Madagascar’s coral assemblages: Reefs with high coral diversity, abundance, and function associated with marine protected areas
Source: PLoS One. 2022 Oct 20;17(10):e0275017. doi: 10.1371/journal.pone.0275017 (PMC9584525; doi:10.1371/journal.pone.0275017)
Supplement: S7 Fig — Values have been standardized as effect sizes; circles represent mean parameter estimates and lines represent 95% confidence intervals. Filled dots indicate significant mean values (i.e., different from zero). (PDF) [file pone.0275017.s035.pdf]

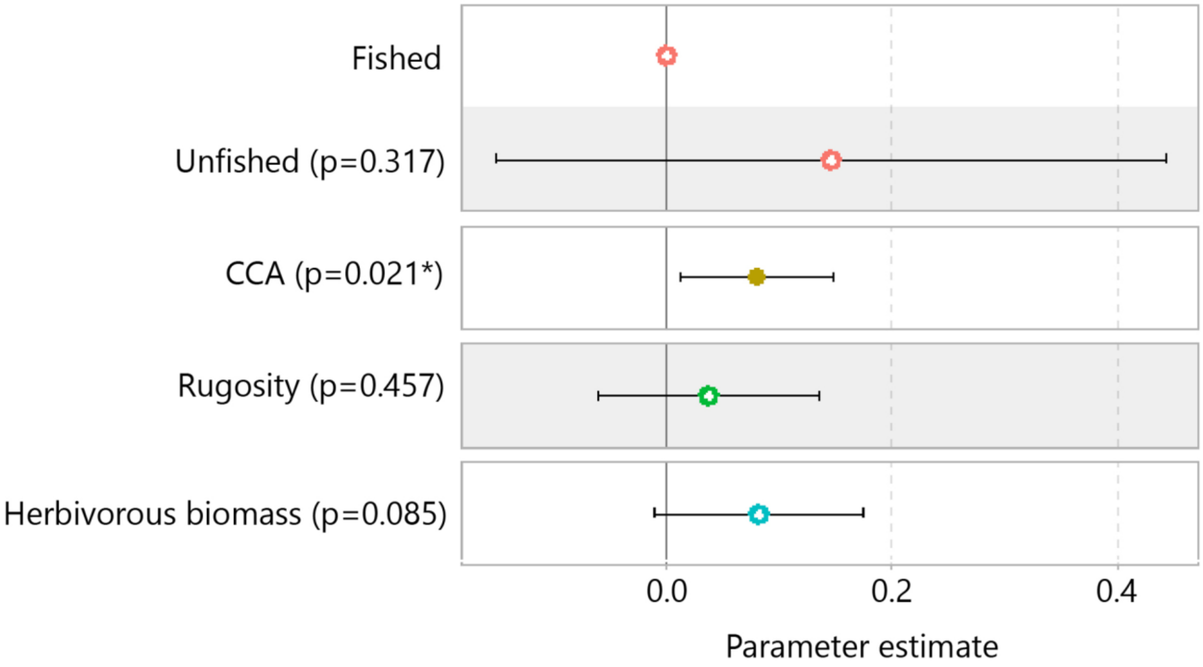

S7 Fig. Mean effects of explanatory variables on the spatial variation in Shannon diversity index. Values have been standardized as effect sizes; circles represent mean parameter estimates, and lines represent 95% confidence intervals. Filled dots indicate significant mean values (i.e., different from zero).
